# Perspective on Multimodal Imaging Techniques Coupling Mass Spectrometry and Vibrational Spectroscopy: Picturing the Best of Both Worlds

**DOI:** 10.1021/acs.analchem.0c04986

**Published:** 2021-04-15

**Authors:** Stefania Alexandra Iakab, Pere Ràfols, Xavier Correig-Blanchar, María García-Altares

**Affiliations:** † Rovira i Virgili University, Department of Electronic Engineering, IISPV, 43007 Tarragona, Spain; ‡ Spanish Biomedical Research Centre in Diabetes and Associated Metabolic Disorders (CIBERDEM), 28029 Madrid, Spain

## Abstract

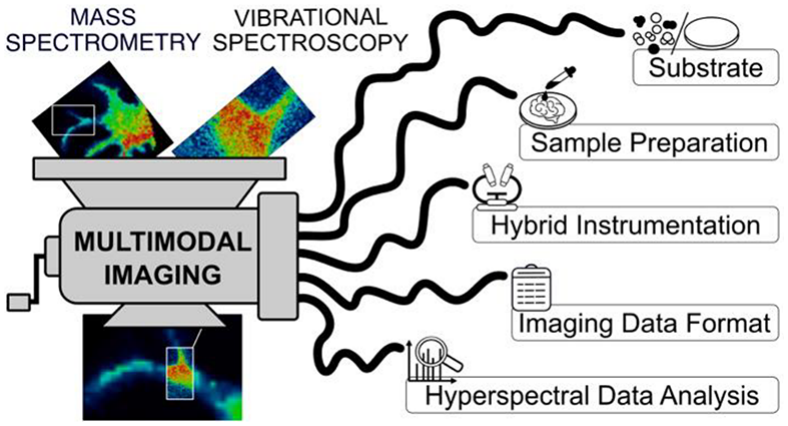

Studies on complex biological phenomena often combine two or more imaging techniques to collect high-quality comprehensive data directly *in situ*, preserving the biological context. Mass spectrometry imaging (MSI) and vibrational spectroscopy imaging (VSI) complement each other in terms of spatial resolution and molecular information. In the past decade, several combinations of such multimodal strategies arose in research fields as diverse as microbiology, cancer, and forensics, overcoming many challenges toward the unification of these techniques. Here we focus on presenting the advantages and challenges of multimodal imaging from the point of view of studying biological samples as well as giving a perspective on the upcoming trends regarding this topic. The latest efforts in the field are discussed, highlighting the purpose of the technique for clinical applications.

A picture is worth a thousand words, especially when it is a molecular image that can shed light on the composition, function, and heterogeneity of a biological tissue. However, one picture might not be enough to fully characterize complex biosystems. That is why multimodal imaging platforms have emerged in the past decade as a coordinated combination of multiple imaging techniques to find solutions in fields as diverse as plant-based renewable energy, microbiology, clinical medicine, and forensics.^1−18^ Multimodal imaging provides a set of information which characterizes biological samples from two aspects: quantitative and qualitative molecular information and molecular distribution at both high and low spatial resolution. The coupling of imaging modalities started with optical microscopy techniques such as classical histopathology, fluorescence imaging, and immunohistochemistry being combined with molecular imaging techniques such as infrared or Raman spectroscopy imaging and mass spectrometry imaging (MSI).^1,2,19−24^ The classical histology techniques have a major limitation which impedes comprehensive analysis of biological samples: they use labels for targeted imaging.^23,25^ This approach involves monitoring a reduced number of compounds (usually two or three proteins) because the chemical stains, immunohistochemical tags, or other labels used for imaging are limited. Additionally, molecular discovery is unfeasible as the use of labels requires rational design of targeted strategies based on specific binding between known molecules. Nevertheless, creative workflows that combine classical histology and label-free techniques can yield valuable information that provide insight into complex biological systems.

Mass spectrometry imaging (MSI) and vibrational spectroscopy imaging (VSI) techniques are common choices for label-free imaging when studying biological tissues. They complement each other in terms of spatial and chemical information, and the limitations of one are complemented by the strengths of the other, so spatial resolution images are better and unique molecular formulas can be identified more effectively. The main complementary characteristics of the two techniques are that VSI can achieve subcellular spatial resolution while MSI is generally used for measuring larger areas and morphologies, and that MSI collects specific molecular information (molecular weight of the ion divided by its charge, *m*/*z*) while VSI adds information about the abundance and specificity of chemical families instead of individual compounds (type of chemical bonds, heavy atoms, *etc.*). Both MSI and VSI have matured greatly in terms of instrumental developments in recent decades. The most commonly used spectrometry techniques for multimodal imaging are matrix-assisted laser desorption/ionization (MALDI), surface-assisted laser desorption/ionization (SALDI), desorption electrospray ionization (DESI), secondary ion mass spectrometry imaging (SIMS), and laser ablation-inductively coupled plasma (LA-ICP). The most commonly used vibrational spectroscopy techniques, on the other hand, are Raman spectroscopy, surface-enhanced Raman scattering (SERS), Fourier-transform infrared spectroscopy (FTIR/IR), and attenuated total reflection-Fourier transform infrared spectroscopy (ATR-FTIR).

## Mass Spectrometry Imaging vs. Vibrational Spectroscopy Imaging

We have summarized in the Supporting Information the fundamentals, characteristics, sample preparation, data acquisition, and data analysis for the most common MSI and VSI techniques used in the latest multimodal imaging applications. Furthermore, Table S1 provides specificities of each technique (lateral resolution, typical spectral range, sample requirements, imaging advantages and challenges). Analysts should keep in mind the following aspects when choosing molecular imaging techniques.

### Sample Requirements

Biological samples (usually tissues, plants and cell cultures) have to comply with the instruments’ probing chamber. For all MSI techniques, samples must be vacuum compatible except for DESI and for a few MALDI sources, where measurements are done in atmospheric conditions. Similarly, all VSI techniques operate in air, however Raman and ATR-IR can also examine samples in liquid. Each technique has their own requirements regarding sample properties (as reflected in Table S1): polarity, surface roughness, thickness, *etc.* Nevertheless, sample preparation for multimodal imaging can be compatible.

### Molecular Information

**Figure 1:**
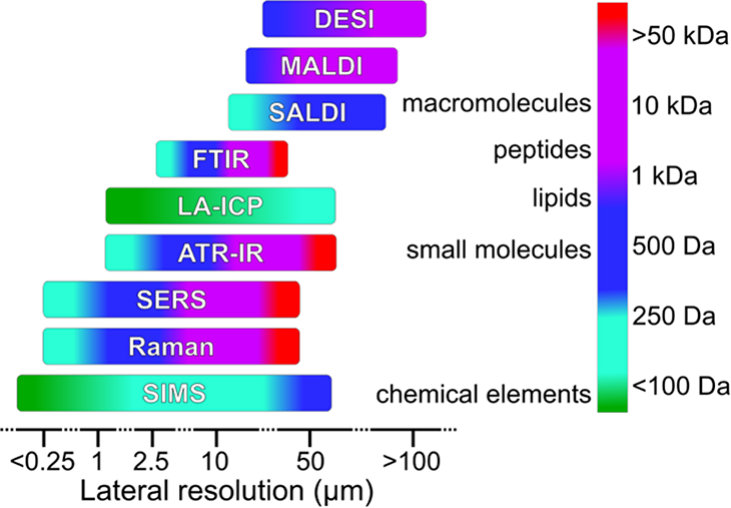
Spatial resolution vs. molecular mass range for MSI and VSI techniques.

In MSI, molecules are detected as ions and, depending on the mass spectrometer and the ionization source, different kinds of molecular classes can be analyzed: DESI and MALDI (both soft ionization techniques) are generally used for lipids, peptides, and proteins; SALDI for metabolites, small molecules and lipids; SIMS (a hard ionization technique) for biologically relevant elements, small molecules, metabolites, and sometimes lipids; and LA-ICP for inorganic compounds such as cations, metals, and other biologically relevant elements (Figure 1). However, identifying molecules in MSI is sometimes a challenge: isomers, enantiomers, isobars, and neutral molecules cannot be recognized. On the other hand, VSI methods examine the vibrations of chemical bonds which provide the fingerprint signature of a molecule. In this case, ATR-IR, FTIR, Raman, and SERS examine the abundance, structure, and conformation of all biomolecules: lipids, proteins, nucleic acids, sugars, DNA, *etc.* Therefore, the complementary information on MSI and VSI is key for uncovering molecular mysteries.

### Type of Experiment

Targeted analyses pinpoint specific molecules by looking for their molecular fingerprint or their expected ion formation (the molecular weight of their specific adducts, isotopes, and fragments). Each technique has its own strategy to achieve this: MALDI uses specific ionizing agents (often called “matrixes”) like 2,5-dihydroxybenzoic acid for peptides or 9-aminoacridine for lipids, SALDI uses nanomaterials to obtain a clean mass spectrum to distinguish small molecules and metabolites, while SERS employs functionalized nanomaterials to enhance the Raman signal of specific molecules. All these approaches demand careful design and sometimes complex sample preparation which are rarely compatible with multiple measurements on the same sample. On the other hand, untargeted analyses need high specificity techniques which observe many molecules simultaneously in order to establish molecular changes and identify key components. In this case MSI techniques are preferred; however, VSI methods can offer complementary information.

### Required Spatial Resolution

Based on the sample size and morphology, different imaging experiments require different spatial resolution. For example, larger samples (tissues, plants) are frequently imaged at lower resolution (over 50 μm per pixel) using techniques such as DESI, MALDI, and FTIR,^3−5,7,10^ while smaller samples (cells, bacteria) are analyzed using high-resolution methods like SIMS, LA-ICP, Raman, and SERS^9,14,15,18^ that can achieve submicrometer resolution. In multimodal imaging, one technique can be used to obtain a full scan of the sample and another to “zoom in” on a morphologically or compositionally interesting area. Fortunately, both MSI and VSI can be used for high- or low-resolution imaging as depicted in Figure 1.

## Advantages of Multimodal Imaging

MSI and VSI can be combined to overcome the limitations of one technique and complementing it by the advantages of the other. This combination has been applied in various imaging studies (as illustrated in Table S1 in the Supporting Information): animal brains (mouse,^7,8,13^ rat,^4,26^ and hamster^3^), cell cultures,^14,16^ bacterial colonies,^9,15,18^ liver metabolism,^1,2^ cancerous tissues,^5,10,17^ fingermarks,^12^ and even plants (perennial grass^11^ and maize leaves^6^). Label-free multimodal imaging is a new holistic approach which will impact analytical chemistry, especially the “omics” sciences, for two main reasons: (1) the imaging techniques provide complementary information that identifies molecules and validates results and (2) the spatial and spectral information have boosted value.

### Complementarity

The information about the lipid content from MSI can be complemented by the protein information provided by VSI.^1,2,13,17^ Even though MALDI-MS is traditionally used for detecting proteins, it also detects lipids when using an organic matrix that facilitates lipid ionization (e.g., alpha-cyano 4-hydroxy cinnamic acid^13^); the lipid signal is also specific to Raman and can inform about their level of saturation, as it is frequently used to analyze lipid droplets, layers, and membranes.^22^ On the other hand, the IR spectra provide specific information about proteins: their composition and secondary structure. For example, Neumann *et al*. used two analytical methods to obtain the chemically specific and spatially resolved information necessary to characterize the chemical heterogeneity of the hippocampus from adult rat brains: the average IR absorption spectrum revealed morphological differences in the protein composition of the different segments within the hippocampus (shift between the amide I and amide II bands), while the average mass spectrum showed significant spatial differences among several phosphatidylcholine lipid species (PC32:0, PC34:1, PC36:4, and PC38:4).^4^ Another complementary function of multimodal imaging is to facilitate the identification of macromolecules, such as polymers. For example, combined information from ATR-IR and MALDI was used to identify condom brands on the basis of their composition: the matrix-solvent combination used for MSI failed to show the presence of polydimethylsiloxane (present in condom lubricants) in all samples, so Raman was used to identify it using the characteristic 1258 cm^–1^ band.^12^ Multimodal techniques have also been used to monitor molecular biosynthesis processes in plants. Burkhow *et al*. monitored the conversion of phytoenes to carotenoids directly in maize leaves and how the rate of this conversion changed when they silenced one of the genes involved in the biosynthesis. Raman described the spatial distribution of carotenoids, but due to the lack of Raman signal to detect phytoenes, SALDI was chosen to monitor phytoene accumulation when they silenced the chosen gene.^6^ In another study, combined information from SERS and LA-ICP-MS demonstrated that hybrid nanoparticles penetrate fibroblast cells differently from standard silica nanoparticles.^14^ The SERS spectra reflected the interaction of several amino acids of the side chains of proteins with the nanoparticles’ surface, while LA-ICP images revealed the cellular uptake of silica nanoparticles with a silver core by monitoring the stable isotope ^107^Ag.^14^ This application promoted the use of multimodal imaging for very much needed qualitative and quantitative nanotoxicology studies.

From the viewpoint of data analysis, multimodal imaging data sets offer a plethora of possibilities for creating both identification and validation algorithms and strategies. The heterospectral data, colocalized mass spectra and vibrational spectra, can be used to identify small variations (*e.g.*, highlighted through PCA or clustering analyses) that represent significant differences between physiologically similar regions within tissues.^3,4^ One study used PCA on a multimodal data set to identify the spatial distribution of different molecular classes: the antibiotic compounds quinolones and quinolines from bacterial biofilms.^18^ The synergistic data processing of both VSI and MSI data highlights small changes that a single technique often overlooks. For example, fused MALDI and Raman images of rat hippocampus revealed differences in protein (bands 1250 cm^–1^, 1550 cm^–1^, and 1680 cm^–1^) and lipid (ions *m*/*z* 772.52, *m*/*z* 789.54, *m*/*z* 820.52) composition between physiologically similar regions, information otherwise inaccessible by individual images due to lack of specificity or spatial resolution of Raman and MALDI images, respectively.^4^ Small details are often crucial for correct spectral interpretation and the correct identification of molecules. The identification of molecules can also be validated with multimodal imaging data sets, as VSI and MSI are orthogonal methods (*i.e*., methods that use fundamentally different principles). For example, the DESI-MSI and Raman imaging correlation map was used to assign structural features to individual molecular species: the multimodal analysis identified the molecular weight (from MS) and the saturation levels (from Raman) of specific lipids (phosphatidylcholines and phosphatidylethanolamines) involved in the myelination process in multiple sclerosis animal model and human brain samples.^7^

### Boosted Spatial and Spectral Information

Due to the limited spatial resolution of some molecular imaging techniques (MALDI and DESI), information-rich MS images are often colocalized with higher resolution VS images^5,11,15,16^ or *vice versa*.^1,2^ In this way, precise localization and molecular identification can be achieved simultaneously. For example, a large area of crop leaves was mapped with LDI-MSI (100 μm lateral resolution) which pinpointed the regions of interest for higher resolution images collected with Raman (∼0.6 μm lateral resolution) and SIMS (∼2 μm lateral resolution) imaging.^11^ In this way high-quality spectra and high-resolution images assigned intracellular globular structures to hemicellulose-rich lignin complexes in perennial grass (*Miscanthus x giganteus*). The coregistration of the two types of image was also ingeniously used to categorize tissue types. This time larger areas were collected by VSI in order to guide and select the regions of interest for MSI. Specifically, FTIR microscopy was used to automatically guide high-resolution MSI data acquisition and interpretation (without prior histopathological tissue annotation) through *k*-means segmentation algorithms to separate tumors from healthy tissues in mouse brains.^5^ Another study used the histopathological analysis of Raman spectra as a guide for MALDI analysis, which differentiated between healthy and altered epithelial growth from a larynx carcinoma sample.^17^ In this study, MS revealed many overexpressed ions which are associated with tumor markers (*e.g.*, sphingomyelins, several phosphatidylcholines, and a high content of glycerophospholipids). So, changes occurring within small areas of tissues could be monitored with both VSI and MSI, and biomarkers were identified.

## Challenges of Multimodal Imaging

Multimodal imaging is a complex analytical tool which involves a variety of high-end instrumentation and software, so it is challenging from two points of view: (1) the experimental workflow and (2) the data processing algorithms.

### Experimental Challenges

Multimodal imaging workflows require multiple optimization steps in each modality to obtain maximum output while maintaining sample and substrate compatibility. Therefore, sample preparation, coregistration strategies that depend on sample preparation, and acquisition parameters have to be optimized for all instruments.

Fortunately, MSI and VSI sample preparation is compatible as the starting point is the same: the biological samples are placed on a substrate. However, the substrate (and thus the analyzed sample) is not always the same for the two techniques. Raman and IR typically use calcium fluoride, but there are other working options such as microscope slides,^1,6,7^ ITO slides,^8,10,13,16,17^ BVDA gelatin lifters,^12^ low emission glass slides,^4^ gold-coated slides,^2,5^ silicon wafers,^11,15,18^ and sterile coverslips.^14^ For MSI, the substrates also vary according to the technique and application: ITO slide,^3,8,10,13,16,17^ MALDI plate,^12^ custom-made SIMS plate,^9^ low-emission glass slide,^4^ gold-coated slide,^2,5^ packing tape,^6^ silicon wafer,^1,11,15,18^ magnesium fluoride slide,^7^ and sterile coverslips.^14^ Choosing a substrate that worked for both imaging techniques soon became an asset, and several studies applied multimodal imaging using the same sample preparation and the same sample.^2,4,5,10,11,13,16,17^ One such substrate is the ITO-coated glass slide, on which cells,^16^ mouse brain,^4,5,13^ mammary tumor,^10^ and larynx^17^ sections are deposited right after cryosectioning. Generally, the Raman measurements (which are nondestructive) are done first, and then the matrix is deposited on the substrate for MALDI analysis. Silicon wafers are another example of a commonly used substrate in SIMS, LDI, and Raman,^11,15,18^ while gold-coated glass slides are used to couple SIMS and synchrotron FTIR and UV spectroscopy.^2^ All these substrates provide excellent spectra in both modalities with minimal preparation and interference in the data acquisition. However, when signal enhancing agents are used (such as organic matrixes for MALDI), the sample cannot be further used for other measurements without removing the agents, which may wash the analytes away. For this reason, one of the most pressing future steps is to develop more multimodal substrates to enhance both the Raman/IR signal and the ionization efficiency for MSI.

When it comes to instrumentation, sample compatibility is as important as choosing the right substrate. For example, biological samples might be formalin-fixed paraffin embedded. This affects both VSI and MSI measurements because the signal from the paraffin overwhelms the spectra from the sample and conceals relevant information.^27,28^ For this reason, the pretreatment of samples (washing, on-tissue digestion, derivatization, *etc.*) has to be compatible with both imaging techniques. On the other hand, fresh frozen or ice embedded samples might generate artifacts in the VSI spectra due to high water content and autofluorescence. It is also important for the sample to be compatible with vacuum or atmospheric conditions. Dannhorn *et al.* studied the sample preparation methods used in various MSI techniques (DESI, MALDI, and SIMS) to create a universal embedding protocol suited for a broad range of specimens.^29^ The hydroxypropyl-methylcellulose and polyvinylpyrrolidone polymer hydrogel outperformed the standard procedures (optimal cutting temperature medium, gelatin, *etc*.), with no interference with MS analysis or histological stains. Although this approach still needs to solve issues such as cold embedding and thaw mounting samples, it also enables using the same sample section for both immunohistochemical staining and MSI.^29^

Before acquisition, however, an additional sample preparation step might be used for easy image registration during data analysis. This step consists of marking the area to be imaged with a mask or specific points that are later used to coordinate the alignment of the molecular images collected. One such strategy aimed to register images by placing a physical mask, known as a fiducial mask, on the sample to assist in locating the raster positions for both Raman and MALDI imaging techniques.^16^

After ensuring compatible sample preparation, acquisition order is paramount. All MSI techniques are destructive so the molecules detected are no longer present in the remaining sample after acquisition. The sample itself may deteriorate greatly (*i.e*., total ablation by the laser, dehydration, chemical degradation, *etc*.). Therefore, when the sample is probed subsequently by another molecular imaging method, the quality of the collected data can be compromised. Neumann *et al.* examined how the IR data is altered when measuring the same sample after MALDI acquisition. The IR image quality was preserved, but a slight red shift in the spectra was associated with increased sample absorbance from the matrix. Other influencing factors were also noted: altered microenvironment, loss of some chemical species, or exposure to atmosphere.^4^ Nevertheless, if the first imaging method is nondestructive, which is the case for all VSI methods, then the following acquisition generates reliable information. As such, the latter approach is the norm (see Table S2).

To sum up, for both imaging techniques the analyst must ensure that (1) the acquisition parameters are compatible with obtaining the required spatial and molecular information, (2) the acquisition time is minimal (if sample viability is a concern), and (3) the images are suitable for postacquisition image coregistration to avoid resolution discrepancy.

### Data Processing Challenges

**Figure 2:**
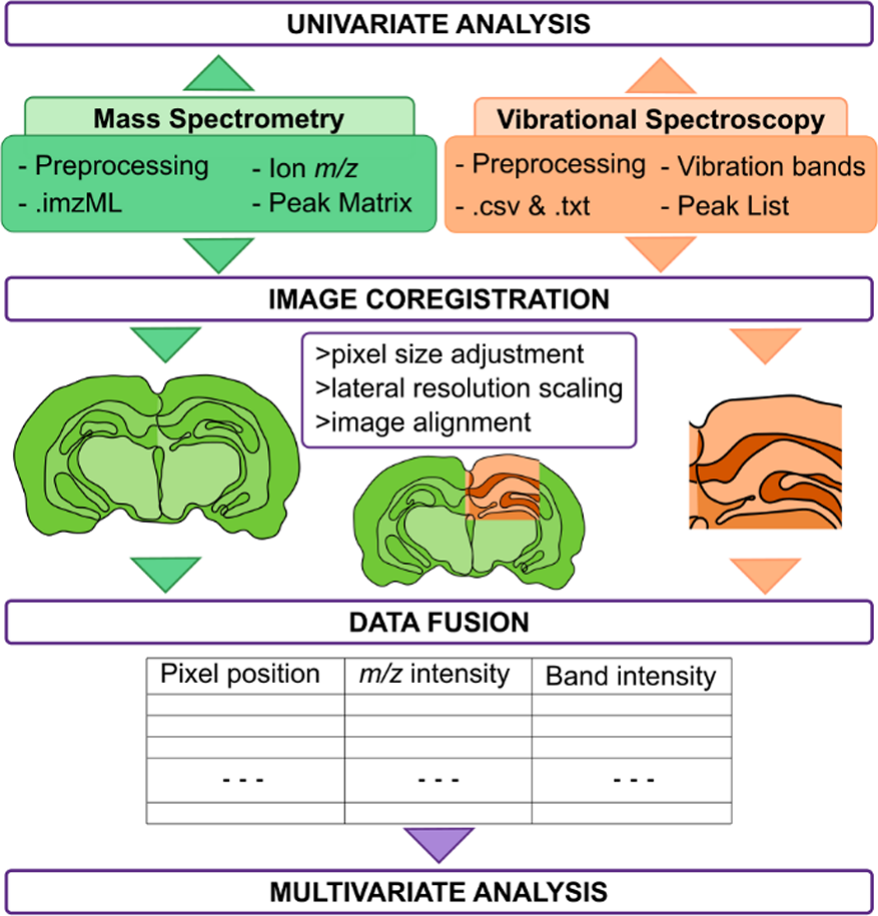
Generalization of the image and data fusion workflow combining one MSI and one VSI technique.

Data processing and analysis strategies are needed to extract the maximum chemical information. In the case of multimodal imaging, these strategies strongly depend on the experimental workflow. Multimodal imaging data is a collection of two datacubes which contain different spectral information but often similar spatial information. Generally, the preprocessing step is done separately for each datacube, in accordance with the techniques’ necessities. This involves using different software for each technique and possibly different algorithms for similar signal processing procedures. Figure 2 illustrates a generalized multimodal data processing workflow for MSI and VSI images, where the preprocessing step is done separately for each image followed by the data analysis step which consists of both image and data registration. Several data analysis approaches have been applied to multimodal data sets. The two prominent strategies are image registration and image or data fusion.

**Figure 3:**
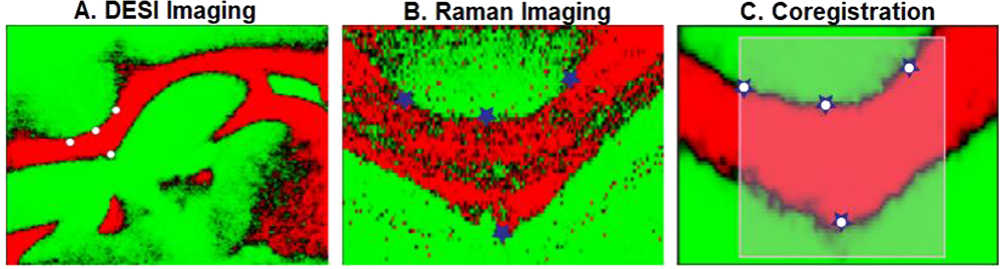
Fiducial markers placed on heterogeneous landmarks in the mouse brain for coregistration by MSI (A) and VSI (B) images. The coregistration results are illustrated in part C where the fiducial markers from MSI (white circles) are superimposed on the ones from VSI (blue stars).^7^ Reproduced with permission from ref (7). Copyright 2017, American Chemical Society.

Image registration, also called coregistration, consists of aligning two different images by selecting control points based on anatomical features present in the sample.^30^ The points (or fiducial markers) are usually selected on optical images, on molecular images with single or multiple ion or band representation, and even on score images from the principal component analysis (PCA). The simplest way to coregister multimodal images is to convert each image (of selected *m*/*z* or bands) into a single-color intensity plot and then overlap the images using the RGB color schemes.^15,16^ This approach is feasible when the images can be aligned and superimposed without distorting the spatial resolution of either image. Another strategy for coregistration used fiducial markers. Pixel-wise coregistration was done with a high-resolution VSI image (for instance Raman, spatial resolution of ∼10 μm) and a low-resolution MSI image (for example, DESI, spatial resolution of ∼50 μm), where the sequential imaging data from the same sample was coregistered using a fiducial marker-based alignment^7^ (see Figure 3). The workflow consisted of placing four markers on each molecular image based on the landmarks on the brain tissue after which an affine transformation was used to coregister the DESI image to match the Raman image. Generally, to correlate the molecular images, more than three marker points should be defined in both images for appropriate registration. A more advanced technique for registering images and sharpening the low-resolution image was based on modeling the distribution of colocalized measurements using partial least-squares (PLS) regression.^30^ Van de Plas *et al.* used molecular images collected with MSI and H&E images from microscopy to predict ion distributions at high spatial resolution in a mouse brain.^30^ This coregistration technique is commonly used in image fusion or data fusion experiments, which not only register the images but also correlate the spectral information.

Data fusion consists of merging the VSI and MSI spectra into one single multidimensional data set called a multimodal datacube. The multimodal datacube describes each pixel with three components: one spatial component giving information on the pixel position and two spectral components (*m*/*z* intensity and band intensity) associated with each pixel, as seen in Figure 2. This allows the spectral information from each pixel in one image to be correlated with the pixels in the other. In this way, the multimodal data set can be used to predict an MSI spectrum in a VSI pixel or *vice versa*, on the basis of the interdependence between VSI and MSI. In the scientific literature, each study tackles data fusion differently.^4,8,10^ Ryabchykov *et al.* fused the spectral data from MALDI and Raman images after interpolating the data in the same spatial grid during coregistration. This data fusion consisted of adjusting the dimensionality and dynamic range of each data set and then merging the two matrixes into one.^8^ Similarly, Neumann *et al.* coregistered the MS and IR images using anatomical features of the individual PCA score images and then fused the data by up-sampling the MS image and down-sampling the IR image to adjust discrepancies between the spatial resolution.^4^ Another data fusion strategy transformed the coordinate system of one imaging modality into the other in order to calculate the mean VSI spectra for regions which exhibit a certain intensity of a MSI peak or MSI fingerprint.^13^ This concept was designed to translate any disease marker information from MSI into the complex Raman fingerprint, information which can be further used as an *in vivo* application of the marker in a diagnostic procedure.^13^

**Figure 4:**
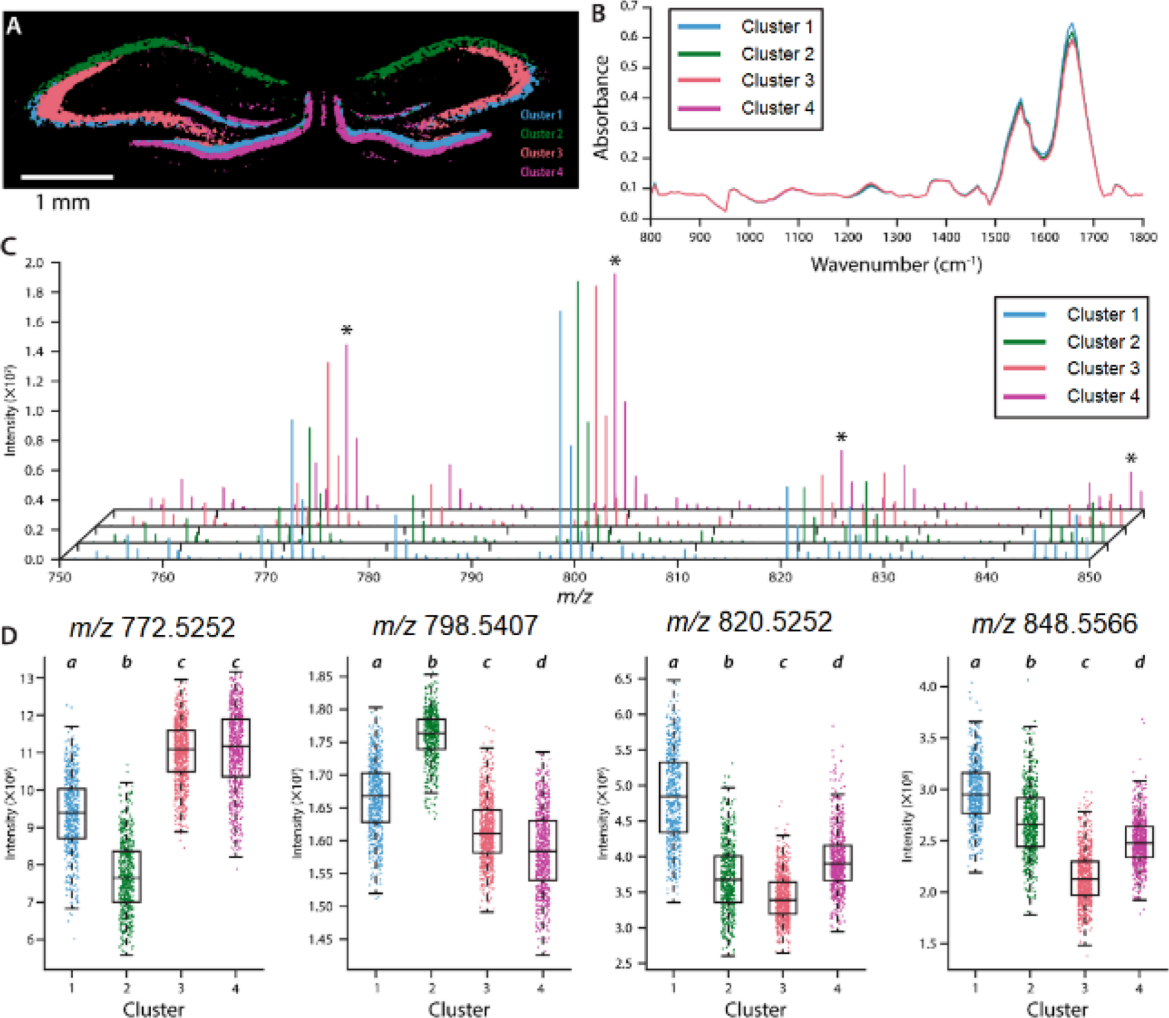
Results of *k*-means clustering of the fused data set. (A) cluster image with each cluster associated with different anatomical regions in the rat hippocampus; (B)average IR absorption spectra per cluster; (C) average MS spectra per cluster; and (D) boxplots illustrating different signal intensities for specific lipid species in each cluster.^4^ Reproduced with permission from ref (4). Copyright 2018, American Chemical Society.

Multimodal data analysis usually consists of multiple multivariate procedures used to discover otherwise buried signals. The algorithms used in the scientific literature include PLS to predict MALDI spectral information from the Raman spectra;^13^ PLS-discriminant analysis (PLS-DA) to classify Raman spectra for differentiating newly formed myelin and normal myelin in remyelination;^7^ maximum margin criterion linear discriminant analysis (MMC-LDA) to determine the most significant mass spectrometry peaks from DESI measurements;^7^ multivariate curve resolution-alternating least-squares (MCR-ALS) to reveal the composition of lipids and their particular localization;^10^ and clustering algorithms for untargeted image segmentation. The most common clustering algorithm is *k*-means clustering,^4,5,7^ but unsupervised hierarchical cluster analysis, which is based on interspectral distances obtained from normalized Pearson’s product-momentum correlation coefficients and Ward’s algorithm, is also used.^3^Figure 4 illustrates the results of a *k*-means clustering on a rat hippocampus, where each cluster is represented spatially (Figure 4A) and has a mean spectrum from each modality (IR absorbance spectra in Figure 4B and MALDI MS spectra in Figure 4C).^4^ The correlated information highlights the differences in lipid (boxplots in Figure 4D) and protein content between clusters, which are associated with different morphological features of the rat hippocampus.

The technical challenges in terms of data processing include the format in which the data is collected and the software used for data analysis. Unfortunately, for imaging experiments there is no universal data format that encompasses both VSI and MSI data. This prevents straightforward data fusion and analysis by a single software package. Recent progress in format compatibility between Raman and SALDI images proposed imzML (the standard format for MSI files) as a template for Raman imaging data as well.^31^ The converter presented in this study transforms Raman imaging data (.txt files) into imzML, and makes it possible to use MSI-specific software for Raman imaging data. This is the first step on the way to creating a common format for all molecular imaging data. On the other hand, multimodal imaging requires several software packages for data export, conversion, processing, and analysis. For example, WinCadence (PHI) was used to generate SIMS images, FlexImaging software (Bruker) was used to generate LDI images, and WITec software was used for Raman data analysis, all in the same multimodal application.^11^ However, some multimodal data analysis approaches use custom-made scripts in R and Matlab,^3,4,13,17^ but data preprocessing is not always done in the same software. For this reason, multimodal imaging data analysis requires a good set of programming skills and/or multiple licensed software.

## Multimodal Imaging and Its Transition to the Clinic

Multimodal imaging is an analytical approach with benefits in the clinical environment that are as yet unexplored. Its high specificity, functional and molecular information, and the label-free aspect of the technique augment the histological data obtained with standard microscopic tools used in the clinics. This approach provides a plethora of possibilities for biomedical applications such as diagnostics, intraoperative guidance, nanotoxicology, and even personalized medicine. For example, modern surgical tools that guide intraoperative interventions already use IR and Raman probing because they are affordable, offer real-time information, and enable molecular profiles to be visualized on the microscale.^32^ Similarly, MS techniques have been used for surgical guidance tools. In particular, iKnife has identified and distinguished between primary and metastatic tumors *in vivo* and *ex vivo*.^33^ However, the imaging possibilities of VSI and MSI are still barely used in clinical medicine. We suggest some possible examples about the use of label-free multimodal imaging that couples VSI and MSI for the applications described below.

### Automatic Classification of Healthy and Diseased Tissues

White light histopathology is the gold-standard for diagnosing cancerous cells from samples of human tissues. However, it relies heavily on the experience of the pathologist. Since experts in histopathology are scarce and expensive to train, several applications^34^ have arisen so that VSI (especially IR imaging) can be used to screen and classify tissues using the approach known as “spectral histopathology.”^24^ These applications are complementary to classic histopathology and aim to reduce the number of samples that pathologists need to examine. There are even commercial applications currently being evaluated in clinical trials that exploit IR spectroscopy in the screening of cancerous tissues, such as esophageal and colon cancer, by the company DynamX Medical (https://www.dynamxmedical.com/). The use of MSI to identify biomarkers emerged from the need not only to distinguish between healthy and cancerous tissues but also to distinguish between cancer types and grades.^20,34^ As such, ambient mass spectrometry imaging analysis revealed biomarkers representative of different types and subtypes of lung cancer with a sensitivity and a specificity of 93.5% and 100%, respectively.^20^ Therefore, multimodal imaging could be used for diagnostic purposes in clinical medicine, where VSI classifies tissues and MSI detects biomarkers of cancer grades, all in an automated procedure with minimum interaction from a pathologist.

### Development of Equipment for the Determination of Surgical Margins

Surgical margins, or margins of resection, are the rims of the apparently healthy tissue around a tumor that has been surgically removed. Normally pathologists examine the surgical margins postoperation to check if tumorous cells are present in the margins of resection. There are devices currently used to characterize surgical margins *in vivo* but they apply MS and vibrational spectroscopy strategies separately: (i) iKnife for electrosurgical dissection of tissues that informs about the tissue lipidomic profiles and discriminates different types of tumors;^33^ and (ii) a hand-held contact Raman spectroscopy probe for the *in vivo*, local detection of cancer cells in the human brain.^35^ Since both methods are hand-held, combining the iKnife and the Raman probe into a single instrument could broaden the possibilities of intraoperative procedures, maximize the analysis possibilities, and give surgeons accurate guidance.

### Cell and Organoid Model Imaging

Understanding cell response and cell–cell interactions is crucial for testing compounds, such as drugs, cosmetics, pollutants, nanoparticles, *etc.*, or for developing therapies and designing *in vitro* organoid models. For example, molecular images collected with SIMS MSI established the silver nanoparticle uptake of Caco-2/TC7 and HT29-MTX cells by analyzing intracellular localization in relation to particle size.^36^ Cell–cell interaction analysis is also crucial when designing organoid models such as the coculture model of the alveolar barrier, which includes several types of cells: alveolar type II epithelial cells, endothelial cells, macrophages, and dendritic cells.^37^ This model was used to discriminate chemical respiratory sensitizers from irritants. Multimodal imaging could be used to monitor oxidative stress, a common consequence of exposure to some toxicants, including nanoparticles, in cells and organoid models through DNA damage and changes in lipid peroxidation.^38^ For this, Raman can monitor DNA conformational changes following several bands between 600 and 1700 cm^–1^,^39^ while MSI can monitor lipid peroxidation by identifying lipid species and their degree of saturation.^40^ Therefore, cell response can be characterized *in situ* as a living system (with VSI) and as a frozen metabolic state (with MSI).

### Noninvasive Testing Protocols

Sweat is a biofluid that contains various excretion products: amino acids, urea, metal and nonmetal ions, metabolites, and xenobiotics (such as drug molecules).^41^ For this reason, noninvasive tests based on sweat composition analysis are becoming popular. MSI approaches were used to monitor illicit drugs from fingerprints^42^ and to observe the detoxification of contaminants and medicines,^41^ and the biomarkers of diseases.^43^ For example, the sweat metabolome analysis by MS revealed that the signal from pilocarpic acid (a metabolite of pilocarpine) and mono(2-ethylhexyl)phthalic acid (a metabolite of the plasticizer bis(2-ethylhexyl) phthalate) can be used to detect cystic fibrosis in asymptomatic infants.^43^ Raman spectroscopy was used to investigate the function of a single human sweat gland and the efficiency of aluminum chlorohydrate, a well-known antiperspirant ingredient.^44^ Future applications of multimodal imaging regarding noninvasive sampling are appealing, especially because multimodal substrates are bioinert and compatible with aqueous samples such as sweat. Additionally, sweat can be collected from the skin surface (for instance, from the palms of the hand) with no need for a particular volume because both VSI and MSI have low limits of detection.

## Perspective and Future Directions of Multimodal Imaging

Sample preparation, coregistration, and data analysis need to be more user-friendly if multimodal imaging is to be used more commonly. That is why standardizing multimodal imaging based on VSI and MSI protocols and developing hybrid instrumentation and software for analysis are key to advancements in the field.

### Universal Sample Substrate

Here we have focused on the imaging part of multimodal techniques, but *in situ* analysis is not always necessary. Investigating urine, saliva, sweat, blood, and even breath does not require spatial information but does often require multiple analytical techniques for accurate diagnosis and even biomarker identification.^45,46^ For this reason, a substrate that is compatible not only with all instruments (both VSI and MSI techniques) but also with all types of sample (liquid, solid, and even gases) is still needed. Solid state substrates based on gold and silicon nanostructures are promising, as they have all the necessary requirements for LDI-MSI and SERS imaging.^47,48^ Because the fabrication processes of these substrates can be automated, and because these types of substrates are compatible with multiple techniques, they are a potential candidate for the standardized multimodal substrate.

Universal sample processing is beneficial not only for multimodal imaging but also for intersectoral and multidisciplinary collaborations. For example, samples prepared in the clinic for histological examination can be stored over time and the collected consortia can later be simultaneously analyzed by nonclinical research groups for different purposes: creation of vast and unbiased spectral reference libraries, disease biomarker identification, development of tissue type classification algorithms, *etc*. Creating a standard coregistration protocol will make it possible to assign two qualitatively different spectra to the same area of a biological sample regardless of the imaging technique used. This will enable fast and accurate data collection over a variety of imaging modalities including MSI, VSI, histology, and even magnetic resonance imaging and other routine clinical imaging techniques.

### Universal Data Format for Imaging

Once imaging data has been collected, the acquisition software should make it possible to export the data in a universal format for imaging. Thus, each imaging data set, regardless of the acquisition technique, can be straightforwardly visualized and analyzed using a single software package. This would facilitate tremendously the development of correlated data analysis algorithms and software which can bring relevant information buried within the multimodal data sets to light. Along these same lines, the use of the imzML format (common for MSI data) has been suggested for Raman imaging data as well.^31^ In fact, the converter presented makes it possible to visualize Raman maps in common MSI software, which provides new opportunities for data analysis. Although the imzML format is missing the nomenclature for Raman measurements such as “Raman Shift” units or diffraction grating definitions (cm^–1^), in the future it could adopt multiple ontology options which could be chosen during data export or conversion. The universal imaging file should have three components: (1) a metadata section that stores all acquisition details such as laser parameters (wavelength, intensity or power, spot size), area specifications (pixel dimensions, step size), specific details of individual methods (integration time for Raman or shots per pixel for MALDI, *etc*.); (2) a coordinates section containing information about pixel position; and (3) a data section with all the spectral information.

### Ultrahigh-Resolution Multimodal Imaging

Multimodal imaging could also push the limits on spatial resolution. In fact, tip-enhanced Raman spectroscopy (TERS) can attain 8 nm,^49^ and optical photothermal infrared (O-PTIR) spectroscopy can reach 100 nm.^50^ TERS molecular images at 8 nm spatial resolution can be achieved through simultaneous collection of Raman spectra and nanotopography information. TERS uses a gold or silver tip which scans the sample surface and, when illuminated, generates a locally amplified electromagnetic field resulting in an enhanced Raman spectra.^49^ O-PTIR has beat the diffraction limit of conventional IR microscopes by modulating the changes of photothermal and photoacoustic effects from an intense IR beam source.^50^ This enables collecting IR spectra in the order of hundreds of nanometers. Moreover, the instrument collects Raman signal simultaneously from the same location, promoting the use of coregistered multimodal imaging. Van De Plas *et al.* demonstrated that fusing rich chemical information images (MALDI MSI) with high spatial information images (microscopy image) can be beneficial from three points of view: (1) the lower resolution image can be sharpened; (2) out-of-sample prediction can be used for acquisitions which have smaller areas; and (3) the enriched spectral information can be used to discover biological patterns otherwise unnoticed.^30^ Therefore, fusing ultrahigh resolution images (from TERS or O-PTIR) with information-rich MS images (such as SIMS) enables the exploration of uncharted territories such as intracellular imaging.

### Hybrid Instrumentation Development

Having a single instrument which combines multiple imaging methods would considerably simplify both experimental and data analysis procedures. In fact, MS instrumentation equipped with an optical microscope is already available on the market as iMScope QT from Shimadzu. A hybrid instrument combining MSI and VSI would analyze the same sample on the same substrate, it would need no coregistration strategy because the coordinate system is identical for both imaging methods, the output data format would be the same, and the data could be analyzed using the same software with no need for multiple data converters. One such multimodal imaging instrument developed in-house already combines atomic force microscopy, IR, and MS to acquire topographical and chemical images at 1.6 μm spatial resolution of PVP/PMMA polymer thin films.^51^ We could also consider the following combinations.

#### Ambient Multimodal Imaging

DESI-MSI combined with Raman spectroscopy using a long working distance objective would enable multimodal imaging in live samples. For example, live bacterial colonies or frozen tissues thawed on a multimodal-compatible substrate could first be sampled with Raman and then immediately sampled with DESI, so after each line acquisition the other modality would measure the same area. The use of an LWD objective could also permit simultaneous acquisition directly from the same pixel if the laser spot size is smaller than the ESI liquid bridge (droplet) and the optical pathway is not hindered by the presence of the capillaries.

#### Three-Dimensional Imaging

SIMS and Raman can be used to obtain 3D profiles of tissues. The ability of confocal Raman spectroscopy to acquire in-depth information and create 3D profiles without damaging the sample is useful for guiding sample discovery.^9^ On the other hand, SIMS is a technique which can be used to collect maps layer by layer or to section (or mill) the sample using the ion beam at the desired depth, previously determined by the Raman profile. Therefore, the fast maps collected with Raman would guide the SIMS analysis, which provides exact information on elements and small molecules.

#### Laser-Based Multimodal Imaging

SALDI and SERS both need a nanostructured agent which interacts with a laser and enhances the collected molecular signal. Therefore, it is reasonable to modify an LDI instrument (such as MALDI) by coupling multiple lasers (UV for MS and visible for Raman) and installing an additional objective or detector (for collecting the SERS spectra). This instrument can explore vacuum-compatible samples such as frozen tissues sputtered with gold or silver nanoparticles, first analyzed by SERS, because it is nondestructive, and then by SALDI. Similarly, a recently presented MALDI instrument that works in transmission mode^52^ can be coupled with an IR laser and detector. This can quickly scan the sample with the IR mode which can help the MALDI acquisition reduce time and increase spatial resolution to the maximum.

### Software Development for Multimodal Data Analysis

Current multimodal workflows collect and preprocess data sets separately and then visualize and analyze single or coregistered images either separately or with data fusion methods. Unfortunately, there is no straightforward way of doing all these data processing steps with the same software. For this reason, software in which the sophisticated computational processes are automatic (such as image registration and data fusion) and users can opt for a friendly interface which requires minimal input are in high demand, especially in clinical medicine where large data sets are routine. For example, LipostarMSI is a software capable of raw data processing, coregistration, manually drawing or importing of regions annotated by pathologists, image visualization, univariate and multivariate image and spectral analysis, and even annotating lipids and metabolites (https://www.moldiscovery.com/software/lipostarmsi/). We foresee an increase in developing such software not only for multimodal MSI and VSI data analysis but also for optical microscopy images, fluorescence imaging, and possibly MRI.

## Conclusion

Multimodal imaging based on MSI and VSI techniques blends the advantages of each modality, specificity, sensitivity, and spatial resolution, and sheds light on details otherwise buried under thousands of pixels. The complete integration of imaging techniques from the point of view of the experimental workflow makes it possible to adopt a multidisciplinary approach to sample processing, which uses not only MSI and VSI but also MRI, histology, and other imaging techniques. As far as multimodal data processing is concerned, universal imaging file formats and standard data processing and analysis workflows will soon be available so that multimodal imaging can be used in various fields. We believe that the progress made in standardizing the multimodal imaging workflow and development of hybrid instrumentation will have considerable impact on research in biology, agriculture, nanotoxicology, pharmaceutics, cosmetics, and especially, clinical applications.

## Supporting Information Available

Detailed description of MSI and VSI techniques, figures which illustrate MSI and VSI working mechanisms, specificities of each MSI and VSI method, and all the multimodal approaches presented in this paper (PDF)

## References

[ref1] Le Naour F.; Bralet M.-P.; Debois D.; Sandt C.; Guettier C.; Dumas P.; Brunelle A.; Laprévote O. Chemical Imaging on Liver Steatosis Using Synchrotron Infrared and ToF-SIMS Microspectroscopies. PLoS One 2009, 4 (10), e7408 10.1371/journal.pone.0007408. 19823674 PMC2757905

[ref2] Petit V. W.; Réfrégiers M.; Guettier C.; Jamme F.; Sebanayakam K.; Brunelle A.; Laprévote O.; Dumas P.; Le Naour F. Multimodal Spectroscopy Combining Time-of-Flight-Secondary Ion Mass Spectrometry, Synchrotron-FT-IR, and Synchrotron-UV Microspectroscopies on the Same Tissue Section. Anal. Chem. 2010, 82 (9), 3963–3968. 10.1021/ac100581y. 20387890

[ref3] Lasch P.; Noda I. Two-Dimensional Correlation Spectroscopy for Multimodal Analysis of FT-IR, Raman, and MALDI-TOF MS Hyperspectral Images with Hamster Brain Tissue. Anal. Chem. 2017, 89 (9), 5008–5016. 10.1021/acs.analchem.7b00332. 28365985

[ref4] Neumann E. K.; Comi T. J.; Spegazzini N.; Mitchell J. W.; Rubakhin S. S.; Gillette M. U.; Bhargava R.; Sweedler J. V. Multimodal Chemical Analysis of the Brain by High Mass Resolution Mass Spectrometry and Infrared Spectroscopic Imaging. Anal. Chem. 2018, 90 (19), 11572–11580. 10.1021/acs.analchem.8b02913. 30188687 PMC6168410

[ref5] Rabe J. H.; Sammour D. A.; Schulz S.; Munteanu B.; Ott M.; Ochs K.; Hohenberger P.; Marx A.; Platten M.; Opitz C. A.; Ory D. S.; Hopf C. Fourier Transform Infrared Microscopy Enables Guidance of Automated Mass Spectrometry Imaging to Predefined Tissue Morphologies. Sci. Rep. 2018, 8, 6361 10.1038/s41598-018-24807-z. 29321555 PMC5762902

[ref6] Burkhow S. J.; Stephens N. M.; Mei Y.; Dueñas M. E.; Freppon D. J.; Ding G.; Smith S. C.; Lee Y. J.; Nikolau B. J.; Whitham S. A.; Smith E. A. Characterizing Virus-Induced Gene Silencing at the Cellular Level with in Situ Multimodal Imaging. Plant Methods 2018, 14, 37 10.1186/s13007-018-0306-7. 29849743 PMC5968576

[ref7] Bergholt M. S.; Serio A.; McKenzie J. S.; Boyd A.; Soares R. F.; Tillner J.; Chiappini C.; Wu V.; Dannhorn A.; Takats Z.; Williams A.; Stevens M. M. Correlated Heterospectral Lipidomics for Biomolecular Profiling of Remyelination in Multiple Sclerosis. ACS Cent. Sci. 2018, 4 (1), 39–51. 10.1021/acscentsci.7b00367. 29392175 PMC5785772

[ref8] Ryabchykov O.; Popp J.; Bocklitz T. Fusion of MALDI Spectrometric Imaging and Raman Spectroscopic Data for the Analysis of Biological Samples. Front. Chem. 2018, 6, 257 10.3389/fchem.2018.00257. 30062092 PMC6055053

[ref9] Morales-Soto N.; Dunham S. J. B.; Baig N. F.; Ellis J. F.; Madukoma C. S.; Bohn P. W.; Sweedler J. V.; Shrout J. D. Spatially Dependent Alkyl Quinolone Signaling Responses to Antibiotics in Pseudomonas Aeruginosa Swarms. J. Biol. Chem. 2018, 293 (24), 9544–9552. 10.1074/jbc.RA118.002605. 29588364 PMC6005435

[ref10] Bedia C.; Sierra À.; Tauler R. Application of Chemometric Methods to the Analysis of Multimodal Chemical Images of Biological Tissues. Anal. Bioanal. Chem. 2020, 412 (21), 5179–5190. 10.1007/s00216-020-02595-8. 32356097

[ref11] Li Z.; Chu L.-Q.; Sweedler J. V.; Bohn P. W. Spatial Correlation of Confocal Raman Scattering and Secondary Ion Mass Spectrometric Molecular Images of Lignocellulosic Materials. Anal. Chem. 2010, 82 (7), 2608–2611. 10.1021/ac100026r. 20205411

[ref12] Bradshaw R.; Wolstenholme R.; Ferguson L. S.; Sammon C.; Mader K.; Claude E.; Blackledge R. D.; Clench M. R.; Francese S. Spectroscopic Imaging Based Approach for Condom Identification in Condom Contaminated Fingermarks. Analyst 2013, 138 (9), 2546 10.1039/c3an00195d. 23486747

[ref13] Bocklitz T. W.; Crecelius A. C.; Matthäus C.; Tarcea N.; von Eggeling F.; Schmitt M.; Schubert U. S.; Popp J. Deeper Understanding of Biological Tissue: Quantitative Correlation of MALDI-TOF and Raman Imaging. Anal. Chem. 2013, 85 (22), 10829–10834. 10.1021/ac402175c. 24127731

[ref14] Drescher D.; Zeise I.; Traub H.; Guttmann P.; Seifert S.; Büchner T.; Jakubowski N.; Schneider G.; Kneipp J. In Situ Characterization of SiO 2 Nanoparticle Biointeractions Using BrightSilica. Adv. Funct. Mater. 2014, 24 (24), 3765–3775. 10.1002/adfm.201304126.

[ref15] Lanni E. J.; Masyuko R. N.; Driscoll C. M.; Dunham S. J. B.; Shrout J. D.; Bohn P. W.; Sweedler J. V. Correlated Imaging with C 60 -SIMS and Confocal Raman Microscopy: Visualization of Cell-Scale Molecular Distributions in Bacterial Biofilms. Anal. Chem. 2014, 86 (21), 10885–10891. 10.1021/ac5030914. 25268906 PMC4221875

[ref16] Ahlf D. R.; Masyuko R. N.; Hummon A. B.; Bohn P. W. Correlated Mass Spectrometry Imaging and Confocal Raman Microscopy for Studies of Three-Dimensional Cell Culture Sections. Analyst 2014, 139 (18), 4578 10.1039/C4AN00826J. 25030970

[ref17] Bocklitz T.; Bräutigam K.; Urbanek A.; Hoffmann F.; von Eggeling F.; Ernst G.; Schmitt M.; Schubert U.; Guntinas-Lichius O.; Popp J. Novel Workflow for Combining Raman Spectroscopy and MALDI-MSI for Tissue Based Studies. Anal. Bioanal. Chem. 2015, 407 (26), 7865–7873. 10.1007/s00216-015-8987-5. 26374565

[ref18] Baig N. F.; Dunham S. J. B.; Morales-Soto N.; Shrout J. D.; Sweedler J. V.; Bohn P. W. Multimodal Chemical Imaging of Molecular Messengers in Emerging Pseudomonas Aeruginosa Bacterial Communities. Analyst 2015, 140 (19), 6544–6552. 10.1039/C5AN01149C. 26331158 PMC4570871

[ref19] Porta Siegel T.; Hamm G.; Bunch J.; Cappell J.; Fletcher J. S.; Schwamborn K. Mass Spectrometry Imaging and Integration with Other Imaging Modalities for Greater Molecular Understanding of Biological Tissues. Mol. Imaging Biol. 2018, 20 (6), 888–901. 10.1007/s11307-018-1267-y. 30167993 PMC6244545

[ref20] Li T.; He J.; Mao X.; Bi Y.; Luo Z.; Guo C.; Tang F.; Xu X.; Wang X.; Wang M.; Chen J.; Abliz Z. In Situ Biomarker Discovery and Label-Free Molecular Histopathological Diagnosis of Lung Cancer by Ambient Mass Spectrometry Imaging. Sci. Rep. 2015, 5, 14089 10.1038/srep14089. 26404114 PMC4585892

[ref21] Das N. K.; Dai Y.; Liu P.; Hu C.; Tong L.; Chen X.; Smith Z. J. Raman plus X: Biomedical Applications of Multimodal Raman Spectroscopy. Sensors (Basel) 2017, 17 (7), 1592 10.3390/s17071592. PMC5539739 28686212

[ref22] Cheng J. X.; Xie X. S. Vibrational Spectroscopic Imaging of Living Systems: An Emerging Platform for Biology and Medicine. Science (Washington, DC, U. S.) 2015, 350 (6264), aaa8870 10.1126/science.aaa8870. 26612955

[ref23] Cornett D. S.; Reyzer M. L.; Chaurand P.; Caprioli R. M. MALDI Imaging Mass Spectrometry: Molecular Snapshots of Biochemical Systems. Nat. Methods 2007, 4 (10), 828–833. 10.1038/nmeth1094. 17901873

[ref24] Bird B.; Remiszewski S.; Kon M.; Diem M. Infrared Spectral Histopathology (SHP): A Novel Diagnostic Tool for the Accurate Classification of Lung Cancer. Lab. Invest. 2012, 92, 1358–1373. 10.1038/labinvest.2012.101. 22751349

[ref25] Gregson C. Optimization of MALDI Tissue Imaging and Correlation with Immunohistochemistry in Rat Kidney Sections. Biosci. Horiz. 2009, 2 (2), 134–146. 10.1093/biohorizons/hzp016.

[ref26] Balbekova A.; Lohninger H.; van Tilborg G. A. F.; Dijkhuizen R. M.; Bonta M.; Limbeck A.; Lendl B.; Al-Saad K. A.; Ali M.; Celikic M.; Ofner J. Fourier Transform Infrared (FT-IR) and Laser Ablation Inductively Coupled Plasma–Mass Spectrometry (LA-ICP-MS) Imaging of Cerebral Ischemia: Combined Analysis of Rat Brain Thin Cuts Toward Improved Tissue Classification. Appl. Spectrosc. 2018, 72 (2), 241–250. 10.1177/0003702817734618. 28905634

[ref27] Dukor R. K. Vibrational Spectroscopy in the Detection of Cancer. In Handbook of Vibrational Spectroscopy; John Wiley & Sons, Ltd.: Chichester, U.K., 2006; 10.1002/0470027320.s8107.

[ref28] Norris J. L.; Caprioli R. M. Analysis of Tissue Specimens by Matrix-Assisted Laser Desorption/Ionization Imaging Mass Spectrometry in Biological and Clinical Research. Chem. Rev. 2013, 113 (4), 2309–2342. 10.1021/cr3004295. 23394164 PMC3624074

[ref29] Dannhorn A.; Kazanc E.; Ling S.; Nikula C.; Karali E.; Serra M. P.; Vorng J.-L.; Inglese P.; Maglennon G.; Hamm G.; Swales J.; Strittmatter N.; Barry S. T.; Sansom O. J.; Poulogiannis G.; Bunch J.; Goodwin R. J.; Takats Z. Universal Sample Preparation Unlocking Multimodal Molecular Tissue Imaging. Anal. Chem. 2020, 92 (16), 11080–11088. 10.1021/acs.analchem.0c00826. 32519547

[ref30] Van De Plas R.; Yang J.; Spraggins J.; Caprioli R. M. Image Fusion of Mass Spectrometry and Microscopy: A Multimodality Paradigm for Molecular Tissue Mapping. Nat. Methods 2015, 12 (4), 366–372. 10.1038/nmeth.3296. 25707028 PMC4382398

[ref31] Iakab S. A.; Semente L.; Garcia-Altares M.; Correig X.; Rafols P. Raman2imzML converts Raman imaging data into the standard mass spectrometry imaging format. BMC Bioinf. 2020, 21, 448 10.1186/s12859-020-03789-8. PMC7547406 33036551

[ref32] Mascagni P.; Longo F.; Barberio M.; Seeliger B.; Agnus V.; Saccomandi P.; Hostettler A.; Marescaux J.; Diana M. New Intraoperative Imaging Technologies: Innovating the Surgeon’s Eye toward Surgical Precision. J. Surg. Oncol. 2018, 118 (2), 265–282. 10.1002/jso.25148. 30076724

[ref33] Balog J.; Sasi-Szabó L.; Kinross J.; Lewis M. R.; Muirhead L. J.; Veselkov K.; Mirnezami R.; Dezso B.; Damjanovich L.; Darzi A.; Nicholson J. K.; Takáts Z. Intraoperative Tissue Identification Using Rapid Evaporative Ionization Mass Spectrometry. Sci. Transl. Med. 2013, 5 (194), 194ra93 10.1126/scitranslmed.3005623. 23863833

[ref34] Willetts K.; Farr L.; Foreman L.; Willetts K.; Farr L.; Foreman L. From Stellar Composition to Cancer Diagnostics. Contemp. Phys. 2019, 60 (3), 211–225. 10.1080/00107514.2019.1645492.

[ref35] Jermyn M.; Mok K.; Mercier J.; Desroches J.; Pichette J.; Saint-arnaud K.; Bernstein L.; Guiot M.; Petrecca K.; Leblond F. Sci. Transl. Med. 2015, 7 (274), 274ra19 10.1126/scitranslmed.aaa2384. 25673764

[ref36] Georgantzopoulou A.; Serchi T.; Cambier S.; Leclercq C. C.; Renaut J.; Shao J.; Kruszewski M.; Lentzen E.; Grysan P.; Eswara S.; Audinot J. N.; Contal S.; Ziebel J.; Guignard C.; Hoffmann L.; Murk A. T. J.; Gutleb A. C. Effects of Silver Nanoparticles and Ions on a Co-Culture Model for the Gastrointestinal Epithelium. Part. Fibre Toxicol. 2015, 13, 9 10.1186/s12989-016-0117-9. PMC4756536 26888332

[ref37] Chary A.; Serchi T.; Moschini E.; Hennen J.; Cambier S.; Ezendam J.; Blömeke B.; Gutleb A. C. An in Vitro Coculture System for the Detection of Sensitization Following Aerosol Exposure. ALTEX 2019, 36 (3), 403–418. 10.14573/altex.1901241. 30791047

[ref38] Manke A.; Wang L.; Rojanasakul Y. Mechanisms of Nanoparticle-Induced Oxidative Stress and Toxicity. BioMed Res. Int. 2013, 2013, 942916 10.1155/2013/942916. 24027766 PMC3762079

[ref39] Sofińska K.; Wilkosz N.; Szymoński M.; Lipiec E. Molecular Spectroscopic Markers of DNA Damage. Molecules 2020, 25 (3), 561 10.3390/molecules25030561. PMC7037412 32012927

[ref40] Sparvero L.; Amoscato A.; Kochanek P.; Pitt B.; Kagan V.; Bayır H. Mass-Spectrometry Based Oxidative Lipidomics and Lipid Imaging: Applications in Traumatic Brain Injury. J. Neurochem. 2010, 115 (6), 1322–1336. 10.1111/j.1471-4159.2010.07055.x. 20950335 PMC3285274

[ref41] Jadoon S.; Karim S.; Akram M. R.; Kalsoom Khan A.; Zia M. A.; Siddiqi A. R.; Murtaza G. Recent Developments in Sweat Analysis and Its Applications. Int. J. Anal. Chem. 2015, 2015, 164974 10.1155/2015/164974. 25838824 PMC4369929

[ref42] Guinan T.; Della Vedova C.; Kobus H.; Voelcker N. H. Mass Spectrometry Imaging of Fingerprint Sweat on Nanostructured Silicon. Chem. Commun. 2015, 51, 6088–6091. 10.1039/C4CC08762C. 25521256

[ref43] MacEdo A. N.; Mathiaparanam S.; Brick L.; Keenan K.; Gonska T.; Pedder L.; Hill S.; Britz-McKibbin P. The Sweat Metabolome of Screen-Positive Cystic Fibrosis Infants: Revealing Mechanisms beyond Impaired Chloride Transport. ACS Cent. Sci. 2017, 3 (8), 904–913. 10.1021/acscentsci.7b00299. 28852705 PMC5571457

[ref44] Chen X.; Gasecka P.; Formanek F.; Galey J. B.; Rigneault H. In Vivo Single Human Sweat Gland Activity Monitoring Using Coherent Anti-Stokes Raman Scattering and Two-Photon Excited Autofluorescence Microscopy. Br. J. Dermatol. 2016, 174 (4), 803–812. 10.1111/bjd.14292. 26574296

[ref45] Bakry R.; Rainer M.; Huck C. W.; Bonn G. K. Analytica Chimica Acta Protein Profiling for Cancer Biomarker Discovery Using Matrix-Assisted Laser Desorption/Ionization Time-of-Flight Mass Spectrometry and Infrared Imaging : A Review. Anal. Chim. Acta 2011, 690 (1), 26–34. 10.1016/j.aca.2011.01.044. 21414433

[ref46] Pahlow S.; Weber K.; Popp J.; Wood B. R.; Kochan K.; Ruther A.; Perez-Guaita D.; Heraud P.; Stone N.; Dudgeon A.; Gardner B.; Reddy R.; Mayerich D.; Bhargava R. Application of Vibrational Spectroscopy and Imaging to Point-of-Care Medicine: A Review. Appl. Spectrosc. 2018, 72, 52–84. 10.1177/0003702818791939. 30265133 PMC6524782

[ref47] Iakab S. A.; Ràfols P.; Tajes M.; Correig-Blanchar X.; García-Altares M. Gold Nanoparticle-Assisted Black Silicon Substrates for Mass Spectrometry Imaging Applications. ACS Nano 2020, 14 (6), 6785–6794. 10.1021/acsnano.0c00201. 32463223

[ref48] Milewska A.; Zivanovic V.; Merk V.; Arnalds U. B.; Sigurjónsson Ó. E.; Kneipp J.; Leosson K. Gold Nanoisland Substrates for SERS Characterization of Cultured Cells. Biomed. Opt. Express 2019, 10 (12), 6172 10.1364/BOE.10.006172. 31853393 PMC6913407

[ref49] Touzalin T.; Joiret S.; Lucas I. T.; Maisonhaute E. Electrochemical Tip-Enhanced Raman Spectroscopy Imaging with 8 nm Lateral Resolution. Electrochem. Commun. 2019, 108, 106557 10.1016/j.elecom.2019.106557.

[ref50] Marcott C.; Kansiz M.; Dillon E.; Cook D.; Mang M. N.; Noda I. Two-Dimensional Correlation Analysis of Highly Spatially Resolved Simultaneous IR and Raman Spectral Imaging of Bioplastics Composite Using Optical Photothermal Infrared and Raman Spectroscopy. J. Mol. Struct. 2020, 1210, 128045 10.1016/j.molstruc.2020.128045. 33859444 PMC8045013

[ref51] Tai T.; Karácsony O.; Bocharova V.; Van Berkel G. J.; Kertesz V. Topographical and Chemical Imaging of a Phase Separated Polymer Using a Combined Atomic Force Microscopy/Infrared Spectroscopy/Mass Spectrometry Platform. Anal. Chem. 2016, 88 (5), 2864–2870. 10.1021/acs.analchem.5b04619. 26890087

[ref52] Niehaus M.; Soltwisch J.; Belov M. E.; Dreisewerd K. Transmission-Mode MALDI-2 Mass Spectrometry Imaging of Cells and Tissues at Subcellular Resolution. Nat. Methods 2019, 16 (9), 925–931. 10.1038/s41592-019-0536-2. 31451764

