# Supplementary material for: Perspective on Multimodal Imaging Techniques Coupling Mass Spectrometry and Vibrational Spectroscopy: Picturing the Best of Both Worlds
Source: Anal Chem. 2021 Apr 15;93(16):6301–10. doi: 10.1021/acs.analchem.0c04986 (PMC8491157; doi:10.1021/acs.analchem.0c04986)
Supplement: Supplementary file 1 — ac0c04986_si_001.pdf [file ac0c04986_si_001.pdf]

Supporting Information for Perspective on multimodal imaging techniques coupling mass spectrometry and vibrational spectroscopy: picturing the best of both worlds

Stefania Alexandra Iakab, Pere Ràfols*, Xavier Correig-Blanchar, María García-Altares

Rovira i Virgili University, Department of Electronic Engineering, IISPV, Tarragona, Spain; Spanish Biomedical Research Centre in Diabetes and Associated Metabolic Disorders (CIBERDEM), Madrid, Spain

Corresponding Author
* Pere Ràfols
e-mail: pere.rafols@urv.cat

Table of Contents

Mass Spectrometry Imaging Methods **2**

Figure S1. Schematic illustration of different ionization mechanisms. **4**

Spectroscopic Imaging Methods **4**

Figure S2. Vibrational spectroscopy methods. **8**

Table S1. Specificities of the MSI and VSI methods used in (multimodal) imaging. **9**

Table S2. Multimodal imaging approaches from the last decade **10**

References **15**

Mass Spectrometry Imaging Methods

Fundamentals. In mass spectrometry each ionized molecule is detected as a mass-to-charge ratio (m/z) and then represented in a mass spectrum. In mass spectrometry imaging, each pixel over an area is represented by a spectrum, and a data cube of pixel position and spectral information (mass spectra spatially correlated with sample morphology) is generated. With this kind of dataset, the position of each m/z can be represented as a map of intensities. Each MSI technique has a different approach to ionizing molecules (illustrated in Figure S1). Readers can refer to Norris et al.[1] for detailed information about the full MSI workflow and Wang et al.[2] for more information about various MSI techniques.

Characteristics. MSI provides abundant high-quality biological information regarding molecular composition and distribution. The chemical information obtained from biological tissues encompasses all classes of biomolecules: small metabolites, medium-sized molecules such as lipids, and large biomolecules such as proteins and polymers, and also biologically relevant elements (in the case of SIMS). MSI techniques are highly specific: depending on the mass analyzer (Time of flight - TOF, Orbitrap or Fourier transform ion cyclotron resonance - FTICR, *etc.*), the spectral resolution can offer mass accuracy better than 1 ppm (accurate to four decimal places) and so can identify ions – single or multiple charged isotopes, adducts and fragments – by their mass-to-charge ratio (m/z).[1] For accurate identification, especially in untargeted studies, tandem MS (MS/MS) analysis is often used.[2] However, MS cannot identify molecules with the same molecular weight, such as isomers and enantiomers, and is not suitable for analyzing neutral molecules or the secondary structure of proteins. The typical lateral resolution in MSI for imaging biological tissues starts from 10-20 μm, but, depending on the technique, can be as low as 0.02 μm (in the case of SIMS).[2] Figure 1 from the main text illustrates the resolution and the molecular information range for common MSI techniques used in multimodal imaging.

Despite being such powerful tools, these techniques have some limitations. Generally, MSI instruments are expensive, they require vacuum-compatible samples (except for DESI), the mass range detection is limited (depending on the type of ionization hard vs. soft ionization), and some methods are constrained to reduced lateral resolution (10 µm for MALDI and 40 µm for DESI due to heterogeneous co-crystallization, analyte delocalization, the spray tip-to-surface distance, and the spray tip and nebulizer orifice diameters).[1,3–5] Lastly, the MSI data collected demands large storage capabilities and powerful computational tools for data preprocessing and analysis.

Sample preparation. Sample preparation is a critical step for any imaging technique. Normally biological samples for mass spectrometry imaging are fresh-frozen tissues[6] although some applications have reported the use of paraffin embedded tissues.[7,8] The typical sample processing before analysis consists of: tissue sectioning, tissue section handling, choosing the right ionization agent and solvent, deposition of the ionization agent, sample transportation and storage.[1] Sometimes, extra steps are included, such as deparaffinization or other specific tissue treatments (e.g. on-tissue washes, enzymatic digestion, chemical derivatization, *etc.*) when necessary.[1]

Data acquisition. The typical MS image measurement starts with the selection and optimization of the acquisition parameters such as mass range, mass resolution, laser power, laser spot size, number of shots per pixel, pixel size, and area of acquisition, but it also includes mass analyzer calibration. Optimizing acquisition parameters is crucial for a successful experiment, as the sensitivity of MSI depends strongly on lateral resolution and ionization efficiency while sample viability can suffer during long experiments.[2] To avoid the acquisition of big datasets in the range of tens to hundreds of gigabytes, all parameters should be optimized to preserve the image quality while also minimizing data size: fewer pixels, precise area of measurement, reduced mass range, *etc.*

Data analysis. The raw data collected from MSI studies goes through several preprocessing algorithms to ensure high quality MS images and optimal statistical analysis. Usually, MSI data consists of a large volume of mass spectra that presents experimental variability – such as chemical

noise and mass spectra shifts – due to sample preparation and small changes during image acquisition. Preprocessing algorithms improve image reconstruction and spectral quality. Rafols et al. described in great detail each step in the general pipeline of preprocessing MSI data.[9] After preprocessing, the data is usually analyzed first by the univariate analysis – or simply spatial visualization – of one specific peak (or ion), and then by more sophisticated analysis such as supervised or unsupervised multivariate analysis. Rafols et al. outlined all aspects necessary for powerful bioinformatics tools reviewing data handling strategies with both commercial and open-source software.[9] Fortunately the mass spectrometry imaging community developed a common data format called imzML,[10] which facilitates progress in MSI processing algorithms, as well as inter-laboratory collaborations in which instruments are not from the same manufacturer.[11]

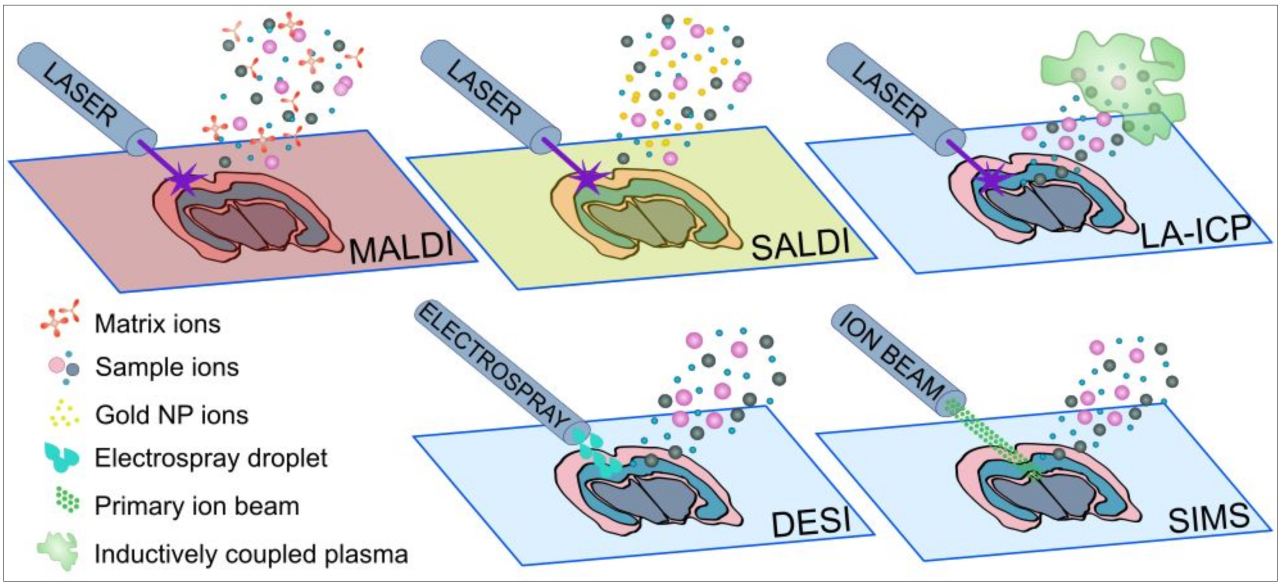

Figure S1. Schematic illustration of different ionization mechanisms.

Spectroscopic Imaging Methods

Fundamentals. Vibrational Spectroscopy is based on light interacting with molecules from a sample. Specifically, the recorded spectrum represents a collection of the molecular vibrations of chemical bonds from all molecules within the illuminated area of the sample.[12] This information gives the fingerprint signature of the sample. For Raman spectroscopy, the incident light of a fixed

wavelength interacts with the sample and the frequency-shifted scattered light is detected and represented in the Raman shift spectrum;[13] for IR spectroscopy the infrared incident light is absorbed by the sample and the transmitted (or reflected) light is detected, resulting in a spectrum of absorption (Figure S2).[14] Just as for MSI, each pixel of an area of the sample is represented by a single spectrum, while the full image contains a data cube of pixel position and spectral information.

Characteristics. Raman and IR spectroscopy describe the physical and chemical properties of molecules by collecting signals that represent the stretching, bending and rotating vibrations of their chemical bonds. Vibrational spectroscopy is label-free and non-destructive, enabling imaging with lateral resolutions from several mm (for mid-IR) to below 1 μm (for Raman). Hence, Raman can be used for single-cell and intracellular imaging[15] while IR is employed for imaging larger areas on tissues[16]. The resulting spectral information gives valuable insights into tissue organization, secondary structure or molecules, lipid and protein content, cell metabolism, drug delivery and even in-vivo prediction of diseases in clinical research. This information is typically obtained from the strong vibrations of specific bonds: for example, lipid droplets and the myelin sheath of neurons are represented by CH_2 vibrations of lipids and CH_3 vibrations of proteins, respectively.[17,18] The lateral resolution in vibrational spectroscopy depends on the optical configuration of the microscope (objective numerical aperture, NA) and the incident laser wavelength (λ), following the laws of physics and optics: spatial resolution = 0.61 λ / NA.[19] Therefore, submicron lateral resolution is easily accessible for various combinations of laser wavelengths and objectives. However, VSI techniques have some limitations. Raman has low sensitivity, long acquisition time per pixel due to its lack of signal strength (ca. 1 scattered photon in 109 incident photons) and low chemical specificity.[5] SERS signal is more sensitive than Raman but it is substrate dependent with reduced spot-to-spot homogeneity, which limits reproducible imaging experiments.[20] IR techniques present strong interference from water absorption, which impedes data acquisition and analysis.[15]

Sample preparation. Raman samples can be either liquids or solids: fresh frozen (*e.g.* tissues on calcium fluoride) or immersed in liquid (*e.g.* cell cultures in aqueous media). Frozen samples follow a similar procedure to MSI: tissue sectioning, tissue section handling, optional deparaffinization or other specific treatments, transportation and sample storage. The tissue sections are typically placed on calcium fluoride slides because their low refractive index and absorption mean that their natural fluorescence is also low.[21] The case of live samples such as cell cultures requires an immersion objective that permits live-cell analysis in real time.[22,23] On the other hand, IR imaging samples are usually solid (*e.g.* tissue sections [7,8,24,25]), and they need to be placed on transparent slides with low absorption in the IR range, such as IR-reflecting microscope slides or calcium fluoride substrates.. Sample preparation for vibrational spectroscopy can be as simple as choosing the sample substrate which does not interfere with the sample signals. Thus, nanostructured materials such as silver[26] and gold[20,27] nanoparticles have been used as sample substrates, mostly because they are signal enhancing agents but also because of their low interference.

Data acquisition. Raman image acquisition parameters strongly depend on the optical configuration of the microscope and the lasers used with the instrument. Laser parameters (wavelength, power, spot shape, *etc.*), the objective's numerical aperture, the type of measurement (in air or immersed in liquid), the spectral grating (for spectral resolution), and the exposure time are the typical acquisition parameters that need to be optimized for spectroscopy measurements. Due to the weakness of Raman scattering and the intrinsic fluorescence of biological samples, the laser wavelength and the objective have to be chosen properly for each experiment so that the spot size, laser power and exposure time provide the best Raman signal without "burning" the samples.[28] Autofluorescence can be reduced by photobleaching[29] and high-resolution images can be achieved with small laser spot sizes (<1 μm). Unfortunately, these conditions together with high laser power damage samples by the localized heat induced on the sample surface. SERS substrates allow using less powerful lasers[5] which ensure sample viability however, long acquisition times are still a threat.

Additionally, SERS imaging measurements struggle with reproducibility,[5] although nanostructured surfaces with high enhancement and homogeneous distribution of hotspots can overcome this limitation.[20] For IR measurements – in transmission, transflection and ATR modes – the spatial resolution depends on the configuration of the IR instrument (single aperture resolution: 2λ/3, and confocal arrangement resolution: λ/2)[30] and on measuring the right background signal. IR spectroscopy is sensitive to substrate transparency (in transmission mode), sample thickness, and water content (reflected in the spectra by the OH band)..[31] IR measurements are mostly held in atmospheric environments, so sample viability over time is also an issue, which is why both Raman and IR spectroscopy seek substrates that allow the temperature to be controlled.

Data analysis. Data analysis for spectroscopic datasets consists of a pre-processing step that prepares the Raman and IR data for analysis[32]. Lasch described in detail the aims of signal pre-processing for both IR and Raman data: (i) robust and accurate spectra; (ii) comprehensible data for both humans and machines; (iii) outlier and trend removal and (iv) dimensionality reduction.[32] Similarly, Vidal *et. al* highlighted the importance of first removing background, dead pixels, spikes and outliers and then pre-processing the remaining spectral data.[33] This approach eliminates all the unwanted effects during acquisition that are both intrinsic (*e.g.* autofluorescence, cell media or water content, substrate, *etc.*) and extrinsic (*e.g.* detector noise, calibration errors, cosmic rays, laser power fluctuations, *etc.*). Gautam *et al.* described all the common pre-processing algorithms regarding spectral axis alignment, cosmic ray removal, background correction (or baseline removal), smoothing, normalization and outlier removal.[34] However, some data processing steps are specific for each type of data: Raman imaging spectra need to be aligned through wavelength calibration and cleaned of cosmic ray artifacts; IR spectra need to pass a quality test, undergo water vapor correction and finally go through a first or second derivative filter for interpretation.[33] Data processing usually consists of univariate analysis in which the spatial visualization of one specific band generates a heatmap, and then of supervised or unsupervised multivariate analysis which finds important structural information,

image segmentation, and tissue classification.[34] Unfortunately, unlike for MSI data, there is no standard or common file format for VSI data storage and processing.

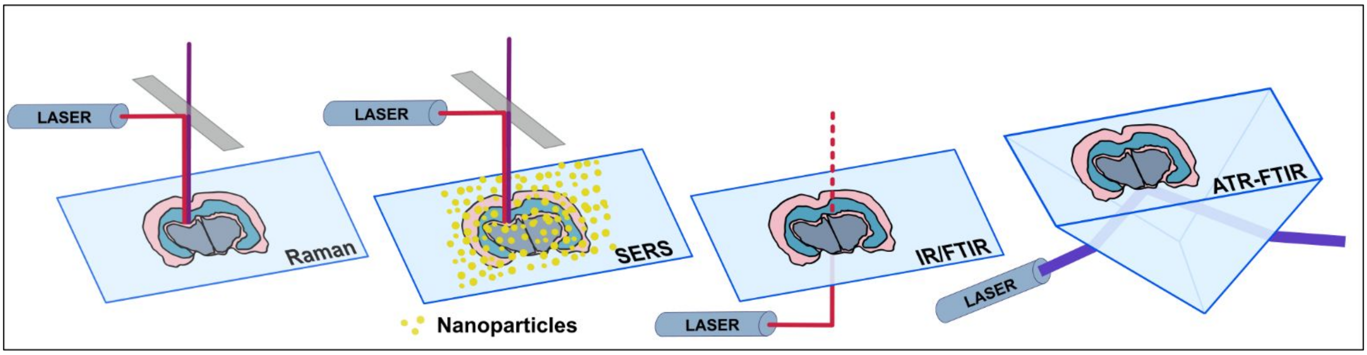

Figure S2. Vibrational spectroscopy methods.

Table S1. Specificities of the MSI and VSI methods used in (multimodal) imaging.

Method	Lat. res.*	Max. lat. res.**	Typical Spectral Range	Sample requirement	Sample preparation	Imaging challenges	Imaging advantages
DESI-MS (*e.g.* Q-TOF)	20-50 µm	10 µm[35]	0-2 kDa	Intermediate polarity to non-polar<br>Flat surface<br>Stable over time	Mounting on non-conductive surface<br>Electrospray composition optimization	Lack of high spatial resolution<br>Electrospray may damage soft samples	Ambiental ionization conditions<br>Soft ionization
MALDI-MS (*e.g.* TOF, QTOF, FTICR)	20-150 µm	1.4 µm[36]	0-70 kDa	Vacuum compatible (dry)<br>Flat surface<br>Stable over time	Mounting on conductive surface<br>Matrix selection and application	Low mass range info missing<br>Matrix effects<br>Analyte delocalization	Wide range of molecules<br>Soft ionization
SALDI-MS (*e.g.* TOF, LTQ-Orbitrap)	20 µm	1.4 µm[36]	0-1 kDa	Vacuum compatible (dry)<br>Flat surface<br>Stable over time	Mounting on conductive surface<br>Nanostructure application	Reproducibility	Analysis in the low mass range (low background)<br>Soft ionization
LA-ICP-MS (*e.g.* Q)	7-40 µm	1 µm[37]	7-250 Da	Vacuum compatible (dry)<br>Flat surface	Mounting on solid support<br>Thin metal layer deposition	Hard ionization<br>"Elemental fractionation"<br>Matrix effects [38]	High precision elemental analysis
SIMS-MS (*e.g.* TOF, QTOF)	1-200 µm	120 nm[39]	0-1 kDa	Vacuum compatible (dry)<br>Flat surface<br>Cryogenic temperature compatible[40]	Mounting on solid support	Low production yield of secondary ions<br>Extensive fragmentation	3D imaging<br>Matrix-free detection<br>High spatial resolution
ATR-IR	18 µm	1 µm[41]	7000–650 cm^{-1}	Flat surface<br>Lower refractive index then ATR crystal	Mounting on ATR crystal<br>Improving contact with ATR crystal by flattening/pressing sample	Water content	Label-free & Non-destructive<br>Measurements in liquid<br>Time dependent analysis<br>Thick samples[42]
FTIR	5-25 µm	diffraction limited ~2.5 µm	4000-700 cm^{-1}	Flat surface<br>Dry sample (low water content)<br>Thin sample	Mounting on low IR absorption transparent substrates	Lack of high resolution<br>Water content	Label-free & Non-destructive<br>Fast acquisition
Raman	0.3-250 µm	diffraction limited ~λ/2 ~250 nm	4000-10 cm^{-1}	Flat surface***<br>Stable over time	Mounting on low refractive index and absorption substrate	Autofluorescence<br>Long acquisition time<br>Lack of specificity	Label-free & Non-destructive<br>Molecular fingerprint<br>Time dependent analysis

SERS	2 µm	diffraction limited<br>~λ/2<br>~250 nm	4000-10 cm^{-1}	Flat surface***<br>Stable over time	Mounting on low refractive index and absorption substrate and nanostructure application<br>Mounting on nanostructured surface	Autofluorescence<br>Long acquisition time<br>Lack of reproducibility	Targeted detection<br>Time dependent analysis

*- lateral resolution range used in multimodal imaging applications presented in this perspective
**- maximum lateral resolution reported in literature
***- The inVia Qontor from Renishaw uses LiveTrack™ focus tracking technology to enable users to analyze samples with uneven, curved or rough surfaces. (https://www.renishaw.com/en/invia-qontor-confocal-raman-microscope--38125)

Table S2. Multimodal imaging approaches from the last decade
(Img. Acq. Order = order of acquisition; Lat. Res. = lateral resolution)

Reference	Method (Img. Acq. Order)	Sample preparation			Acquisition		Data			Results	
		Sample	Substrate + Enhancing agent	Same section	Lat. Res. (µm)	Time	Format/ Software	Processing	Analysis	Specific Information	Synergy
Ahlf 2014[23]	MALDI -TOF (2nd)	Cell cultures	ITO slide + CHCA / DHB / SA	YES	50	N/A	ASCII Bruker	Background correction; matrix peak removal; peak alignment; normalization; PCA	PC correlation; image co-localization	Small molecules; lipids; proteins lipids	50 µm pixel of MALDI corresponds to an entire Raman image; PC score images are fused for colocalization; PCA used to extract chemically informative factors responsible for spatial variation in the spectra from MSI and VSI
	Raman (1st)		ITO slide		1	3h	ASCII WITec	Cosmic ray removal; autofluorescence removal; spectra alignment; normalization; PCA			
Baig 2015[43]	SIMS-QTOF (N/A)	*P. aeruginosa* bacterial biofilm	Si wafer	N/A	200 and 21	N/A	.wiff .img BioMap	Normalization	Univariate analysis (ion map)	Metabolites	SIMS and Raman imaging characterized the spatial distribution of several quinolone quorum sensing molecules and quinoline secondary metabolites across the surface of *P. aeruginosa* at various states of organization. Raman in conjunction with PCA identified broad molecular classes (*e.g.* quinolones and quinolines)
	Raman (N/A)		Si wafer	N/A	0.3	16 min	WITec Matlab	Cosmic ray removal, PCA	Univariate analysis (band map)	Proteins, carbohydrate moieties	

Balbekova 2018[8]	LA-ICP-Q (N/A)	Ischemic stroke rat brain	Immunohistochemistry microscope slides	NO	40	120 µm/s	N/A Imagelab	Normalization	Anchor-points defined spatial alignment; data sets were merged to a hyperspectral datacube; PLS-DA and RDF classification models	Biologically relevant elements	Reliable multivariate analysis: no false-positives or false-negatives were observed for the classification results obtained from models based on the hyperspectral dataset; The RDF classifier built on the combined data sets appeared to be more precise compared to classifiers built on the individual data sets
	FTIR (N/A)		IR-reflecting microscope slides	NO	N/A	N/A	OPUS Imagelab	Baseline correction, normalization, smoothing,		Proteins, nucleic acids, lipids, lipid acyl chain, fatty acids	
Bedia 2020[24]	MALDI-TOF (2nd)	Primary breast carcinoma implanted in the intra-mammary fat path of nude mice	ITO slide + DHB	YES	150	N/A	.imzML Matlab	Peak picking; normalization	x-y orientation correction; image size adjustments by resizing to the same spatial dimensions of the MALDI-MS image (31 × 50); multivariate curve resolution-alternating least squares (MCR-ALS)	Lipids: PC(P-30:1), PA(30:2), PA, PE, PG, PI	The multimodal resolution of the fused images revealed the different lipid compositions associated with specific IR fingerprints, their particular localizations; the limitation of the low spatial resolution of MALDI was compensated for with the higher resolution IR image. The spectra resolved during the analysis of the low spatial resolution images can be used in a second step to recover the distribution maps of the constituents at high spatial resolution by spectral projection
	IR (1st)		ITO slide	YES	25	N/A	.csv Matlab	Baseline correction		Proteins, ester lipids, alcohols, alkyl chains, carboxylic acids	
Bergholt 2018[44]	DESI-QTOF (2nd)	Mouse model brain; human multiple sclerosis brain sections	Magnesium fluoride slides	YES/ NO	20 mice / 50 human	N/A	Matlab	Normalization; ANOVA between control and lesion groups; false discovery rate (FDR) correction; maximum margin criterion linear discriminant analysis (MMC-LDA)	DESI and Raman images coregistered using a fiducial marker-based alignment; pixelwise approach	Lipids	A heterospectral DESI-MS and Raman correlation map enables structural features detected by vibrational spectroscopy to be assigned to individual molecular species detected by mass spectrometry; This technique revealed the subtle and apparently buried Raman peaks; approach to profile the lipidomics of de- and re-myelinated tissue at both the structural and compositional levels
	Raman (1st)		Superfrost Plus adhesive glass slides	YES/ NO	6 mice/ 150 human	34h / 41 min human	WITec	Tissue autofluorescence removal; normalization; partial least-squares discriminant analysis (PLS-DA); *k*-means clustering		Lipids Proteins; DNA	
Bocklitz 2013[45]	MALDI-TOF (2nd)	Mouse brain	ITO slide + CHCA	YES	75	14h	imzML Bruker	Background correction	PLD correlation	Lipids	MALDI signal translated into a Raman fingerprint by multivariate calibration model; predicting the MALDI peak intensity based on the transformed and preprocessed Raman scan
	Raman (1st)		ITO slide		25	3s/ spectrum	WITec	Background correction		Proteins	

Bocklitz 2015[46]	MALDI -TOF (2nd)	Larynx carcinoma	ITO slide + CHCA	YES	25	20h	imzML Bruker	Vector normalization; PCA	Co-registration by rigid registration	Cancer markers; lipidome	Co-registering Raman and H&E image to the MALDI grid; Raman spectroscopy used to determine the tissue type and MALDI imaging used to investigate changes occurring in this tissue type
	Raman (1st)		ITO slide		12.5	21h	WITec	Background correction; vector normalization; PCA		Lipids; proteins; nucleic acids	
Bradshaw 2013[47]	MALDI -QTOF (2nd)	Condom lubricant-contaminated fingermarks	MALDI plate + CHCA	Remaining deposit	N/A	N/A	.wiff .txt Analyst	Spectra recalibration; normalization	Univariate analysis (ion map)	Polymers; endogenous and exogenous compounds	Complementary information to identify condom brands
	ATR-IR (1st)		BDVA gelatine lifter	First lift	18	N/A	Resolutions Pro	N/A	Univariate analysis (band map)	Polymers	
Burkhow 2018[26]	SALDI-LTOQ Orbitrap (N/A)	Gene modified maize leaves	Packing tape + Ag nanolayer	N/A	20	N/A	ImageQuest	N/A	Univariate analysis (ion map)	Phytoene	The accumulation of phytoene by MS imaging agrees with the reduced carotenoid Raman signal within the same area; biochemical changes in carotenoid expression from gene silencing; sensitivity of MS imaging enables the imaging of phytoene and Raman imaging provides high selectivity for downstream carotenoids
	Raman (N/A)		Glass slide	N/A	3	15s	Igor Pro Matlab	Gaussian batch fit and extraction of the ~1520 cm−1 peak amplitudes and maxima; noise quantification within the region of 500–600 cm−1	Univariate analysis (band map)	Vascular bundles; mesophyll and epidermal cells	
Drescher 2014[22]	LA-ICP-MS (N/A)	Mouse fibroblast cells and macrophages	Sterile cover slip + silica-coated Au and Ag nanoparticles	NO	7-8	N/A	Origin ImageJ	N/A	Univariate analysis (ion map)	Number of nanoparticles	Investigation of intra- and extracellular biomolecules; spatially resolved LA-ICP-MSI showed differences in the uptake of nanoparticles into cells and SERS spectra characterized nanoparticle biomolecule interactions; nanoparticles enabled multimodal qualitative and quantitative characterization.
	SERS (N/A)		Sterile cover slip + silica-coated Au and Ag nanoparticles	NO	2	N/A	Matlab	N/A	Univariate analysis (band map)	Biomolecules; culture medium components; pATP reporter	
Lanni 2014[48]	SIMS-QTOF (2nd)	*Pseudomonas aeruginosa* bacterial biofilm	Si wafer	YES	10	N/A	.img BioMap	N/A	Univariate analysis (ion map)	Metabolites	The nanometer-scale spatial resolution provided by Raman is complemented by the chemical specificity of SIMS; correlated detection of at least nine quinolones and additional related metabolites present in biofilms
	Raman (1st)		Si wafer	YES	0.3	16 min	WITec Igor Pro	N/A	Univariate analysis (band map)	Proteins, carbohydrate moieties	

Lasch 2017[25]	MALDI-TOF (3rd)	Hamster brain	ITO slide + DHB	NO	60	N/A	Cytospec	Smoothing; vector normalization; offset correction	*k*-means cluster analysis	Sphingolipids; cholesterol;	Spectra interpretation, band assignment and identification of a very subtle heterospectral correlation pattern; Spectral resolution enhancement procedures, like Pareto-scaling and node attenuation, enabled the assignment of overlapping bands in HSI data
	Raman (2nd)		CaF2 slide	YES	1	1.1h	WITec Cytospec	Baseline correction; cosmic ray removal	Unsupervised hierarchical cluster analysis	Sphingolipids; cholesterol	
	FTIR (1st)		CaF2 slide	YES	25	N/A	Bruker Cytospec	Spectral quality test; vector normalization; first derivative		Sphingolipids	
Le Naour 2009[7]	SIMS-TOF (2nd)	Liver biopsies	Si wafer	NO	1-2	N/A	IonSpec IonImage	normalization	Univariate analysis (ion map)	Lipids	One of the first studies to emphasize the advantages of combining different spectroscopies for investigating in situ the chemical composition of tissues; lipid content characterization and visualization of small lipid droplets
	FTIR (1st)		Glass slide	NO	6-10	N/A	OMNIC	N/A	Univariate analysis (band map)	Proteins, lipids	
Li 2010[49]	SIMS-TOF (2nd)	*Miscanthus x giganteus*, a tall perennial grass	Si wafer	YES	2	N/A	WinCadence	N/A	Univariate analysis (ion map)	Lignin and cellulose fragment ions	Spatially correlated LDI, SIMS and Raman imaging provide high-quality, high-resolution subcellular images of Miscanthus cross sections, and the combination of information from the mass spectrometry and Raman scattering allows specific chemical assignments of observed structures, difficult to assign from either imaging approach alone
	LDI (3rd)		Si wafer	YES	100	N/A	Bruker	N/A	Univariate analysis (ion map)	High MW compounds	
	Raman (1st)		Si wafer	YES	0.65	37 min	WITec	N/A	Univariate analysis (band map)	Lignin and cellulose	
Morales-Soto 2018[50]	SIMS-TOF (N/A)	*P. aeruginosa* bacterial biofilm planktonic cells	Custom aluminium SIMS plate	NO	50	7 min	.imzML MsiReader	PCA	Univariate analysis (ion map)	Targeted alkyl quinolones: PQS, C9-PQS, HQNO, and NQNO	SIMS PCA image results corroborate the relative quantification results and validate the CRM observations, indicating that when exposed to the aminoglycoside tobramycin, *P. aeruginosa* cells undergo a metabolite shift; multimodal imaging for spatial heterogeneity in signaling is possible within a *P. aeruginosa* community
	Raman (N/A)		N/A	NO	0.5	2.5h	WITec Matlab	Removed cosmic ray spikes; PCA	Univariate analysis (band and PCA map)	DNA, proteins, lipids, PQS, AQNO	
Neumann 2018[51]	MALDI-FT-ICR (1st)	Rat brain	Low emission glass slide + DHB	YES	25	N/A	.imzML SCILS Matlab	Normalization; spectra alignment; peak picking	Pan sharpening to sharpen MSI images with higher spatial resolution IR image	Lipids	Data fusion and unsupervised clustering for detecting significant chemical differences; exploring chemical heterogeneity present in the brain
	FTIR (2nd)		Low emission glass slide	YES	5	N/A	.mat Matlab	N/A		Lipids; proteins;	

Petit 2010[52]	SIMS-TOF (2nd)	Liver cirrhosis	Au-coated glass slide	YES	1-2	6 min	IonSpec IonImage	N/A	Univariate analysis (ion map)	Lipids	Investigating biological tissue without any treatment, labeling, or staining of the sample and using a single sample holder; characterization of liver cirrhosis ester lipid species
	FTIR (1st)		Au-coated glass slide	YES	6-10	N/A	OMNIC	N/A	Univariate analysis (band map)	Lipids, proteins, nucleic acids, sugars	
Rabe 2018[16]	MALDI-TOF (2nd)	Mouse brain Human gastrointestinal stromal tumor	Au-coated slide + DHB / PhCCAA	YES	20	18.3h	R, Cardinal package	Peak picking; normalization; baseline correction	Spatially aware segmentation	Lipids; metabolites	FTIR image segmentation guides MSI acquisition; this allows the exclusive acquisition of regions of interest to increase throughput in cases where the acquisition of whole tissue sections at high resolution is impractical or even infeasible
	FTIR (1st)		Au-coated slide	YES	6.5	1h	Matlab	Baseline correction; first derivative; normalization; spectral quality test	*k*-means clustering	Cell groups by molecular feature	
Ryabchykov 2018[53]	MALDI-TOF (2nd)	Mouse brain	ITO slide + CHCA	YES	75	N/A	BrukerFlexData	Noise removal, background correction, and TIC normalization	Before preprocessing and data fusion, the MALDI and Raman spectra were interpolated to the same spatial grid; data fusion; PCA	Lipids	Changes in the lipid content observed by a high correlation of the Raman spectral region with MALDI mass spectra; data fusion increases reliability not only for the spectral features but also for the spatial features present in the data
	Raman (1st)		ITO slide	YES	25	3s/ spectra	WITec	Corrected for fluorescence background and vector normalized		Lipids, proteins and DNA	

References

(1) Norris, J. L.; Caprioli, R. M. Analysis of Tissue Specimens by Matrix-Assisted Laser Desorption/Ionization Imaging Mass Spectrometry in Biological and Clinical Research. *Chem. Rev.* **2013**, *113* (4), 2309–2342.

(2) Wang, T.; Cheng, X.; Xu, H.; Meng, Y.; Yin, Z.; Li, X.; Hang, W. Perspective on Advances in Laser-Based High-Resolution Mass Spectrometry Imaging. *Anal. Chem.* **2020**, *92* (1), 543–553.

(3) Porta Siegel, T.; Hamm, G.; Bunch, J.; Cappell, J.; Fletcher, J. S.; Schwamborn, K. Mass Spectrometry Imaging and Integration with Other Imaging Modalities for Greater Molecular Understanding of Biological Tissues. *Mol. Imaging Biol.* **2018**, *20* (6), 888–901.

(4) Alberici, R. M.; Simas, R. C.; Sanvido, G. B.; Romão, W.; Lalli, P. M.; Benassi, M.; Cunha, I. B. S.; Eberlin, M. N. Ambient Mass Spectrometry: Bringing MS into the "Real World." *Anal. Bioanal. Chem.* **2010**, *398* (1), 265–294.

(5) Masyuko, R.; Lanni, E. J.; Sweedler, J. V.; Bohn, P. W. Correlated Imaging-a Grand Challenge in Chemical Analysis. *Analyst* **2013**, *138* (7), 1924–1939.

(6) Cornett, D. S.; Reyzer, M. L.; Chaurand, P.; Caprioli, R. M. MALDI Imaging Mass Spectrometry: Molecular Snapshots of Biochemical Systems. *Nat. Methods* **2007**, *4* (10), 828–833.

(7) Le Naour, F.; Bralet, M.-P.; Debois, D.; Sandt, C.; Guettier, C.; Dumas, P.; Brunelle, A.; Laprévote, O. Chemical Imaging on Liver Steatosis Using Synchrotron Infrared and ToF-SIMS Microspectroscopies. *PLoS One* **2009**, *4* (10), e7408.

(8) Balbekova, A.; Lohninger, H.; van Tilborg, G. A. F.; Dijkhuizen, R. M.; Bonta, M.; Limbeck, A.; Lendl, B.; Al-Saad, K. A.; Ali, M.; Celikic, M.; Ofner, J. Fourier Transform Infrared (FT-IR) and Laser Ablation Inductively Coupled Plasma–Mass Spectrometry (LA-ICP-MS) Imaging of Cerebral Ischemia: Combined Analysis of Rat Brain Thin Cuts Toward Improved Tissue Classification. *Appl. Spectrosc.* **2018**, *72* (2), 241–250.

(9) Rafols, P.; Vilalta, D.; Brezmes, J.; Cañellas, N.; del Castillo, E.; Yanes, O.; Ramírez, N.; Correig, X. Signal Preprocessing, Multivariate Analysis and Software Tools for MA(LDI)-TOF Mass Spectrometry Imaging for Biological Applications. *Mass Spectrom. Rev.* **2016**, No. 37, 281–306.

(10) Schramm, T.; Hester, A.; Klinkert, I.; Both, J. P.; Heeren, R. M. A.; Brunelle, A.; Laprévote, O.; Desbenoit, N.; Robbe, M. F.; Stoeckli, M.; Spengler, B.; Römpp, A. ImzML - A Common Data Format for the Flexible Exchange and Processing of Mass Spectrometry Imaging Data. *J.*

Proteomics **2012**, *75* (16), 5106–5110.

(11) McDonnell, L. A.; Heeren, R. M. A.; Andrén, P. E.; Stoeckli, M.; Corthals, G. L. Going Forward: Increasing the Accessibility of Imaging Mass Spectrometry. *J. Proteomics* **2012**, *75* (16), 5113–5121.

(12) Prentice, B. M.; Caprioli, R. M.; Vuiblet, V. Label-Free Molecular Imaging of the Kidney. *Kidney Int.* **2017**, *92* (3), 580–598.

(13) Talari, A. C. S.; Movasaghi, Z.; Rehman, S.; Rehman, I. U. Raman Spectroscopy of Biological Tissues. *Appl. Spectrosc. Rev.* **2015**, *50* (1), 46–111.

(14) Bhargava, R. Infrared Spectroscopic Imaging: The next Generation. *Appl. Spectrosc.* **2012**, *66* (10), 1091–1120.

(15) Willetts, K.; Farr, L.; Foreman, L.; Willetts, K.; Farr, L.; Foreman, L. From Stellar Composition to Cancer Diagnostics. *Contemp. Phys.* **2019**, *0* (0), 1–15.

(16) Rabe, J. H.; Sammour, D. A.; Schulz, S.; Munteanu, B.; Ott, M.; Ochs, K.; Hohenberger, P.; Marx, A.; Platten, M.; Opitz, C. A.; Ory, D. S.; Hopf, C. Fourier Transform Infrared Microscopy Enables Guidance of Automated Mass Spectrometry Imaging to Predefined Tissue Morphologies. *Sci. Rep.* **2018**, *8* (1), 1–11.

(17) Cheng, J. X.; Xie, X. S. Vibrational Spectroscopic Imaging of Living Systems: An Emerging Platform for Biology and Medicine. *Science (80-.).* **2015**, *350* (6264).

(18) Freudiger, C. W.; Pfannl, R.; Orringer, D. A.; Saar, B. G.; Ji, M.; Zeng, Q.; Ottoboni, L.; Ying, W.; Waeber, C.; Sims, J. R.; De Jager, P. L.; Sagher, O.; Philbert, M. A.; Xu, X.; Kesari, S.; Xie, X. S.; Young, G. S. Multicolored Stain-Free Histopathology with Coherent Raman Imaging. *Lab. Investig.* **2012**, *92* (10), 1492–1502.

(19) Murphy, D. B.; Davidson, M. W. *Fundamentals of Light Microscopy and Electronic Imaging*; John Wiley & Sons, Inc.: Hoboken, NJ, USA, 2012.

(20) Milewska, A.; Zivanovic, V.; Merk, V.; Arnalds, U. B.; Sigurjónsson, Ó. E.; Kneipp, J.; Leosson, K. Gold Nanoisland Substrates for SERS Characterization of Cultured Cells. *Biomed. Opt. Express* **2019**, *10* (12), 6172.

(21) Fullwood, L. M.; Griffiths, D.; Ashton, K.; Dawson, T.; Lea, R. W.; Davis, C.; Bonnier, F.; Byrne, H. J.; Baker, M. J. Effect of Substrate Choice and Tissue Type on Tissue Preparation for Spectral Histopathology by Raman Microspectroscopy. *Analyst* **2013**, *139* (2), 446–454.

(22) Drescher, D.; Zeise, I.; Traub, H.; Guttmann, P.; Seifert, S.; Büchner, T.; Jakubowski, N.; Schneider, G.; Kneipp, J. In Situ Characterization of SiO 2 Nanoparticle Biointeractions Using BrightSilica. *Adv. Funct. Mater.* **2014**, *24* (24), 3765–3775.

(23) Ahlf, D. R.; Masyuko, R. N.; Hummon, A. B.; Bohn, P. W. Correlated Mass Spectrometry

Imaging and Confocal Raman Microscopy for Studies of Three-Dimensional Cell Culture Sections. *Analyst* **2014**, *139* (18), 4578.
(24) Bedia, C.; Sierra, À.; Tauler, R. Application of Chemometric Methods to the Analysis of Multimodal Chemical Images of Biological Tissues. *Anal. Bioanal. Chem.* **2020**, *412* (21), 5179–5190.
(25) Lasch, P.; Noda, I. Two-Dimensional Correlation Spectroscopy for Multimodal Analysis of FT-IR, Raman, and MALDI-TOF MS Hyperspectral Images with Hamster Brain Tissue. *Anal. Chem.* **2017**, *89* (9), 5008–5016.
(26) Burkhow, S. J.; Stephens, N. M.; Mei, Y.; Dueñas, M. E.; Freppon, D. J.; Ding, G.; Smith, S. C.; Lee, Y. J.; Nikolau, B. J.; Whitham, S. A.; Smith, E. A. Characterizing Virus-Induced Gene Silencing at the Cellular Level with in Situ Multimodal Imaging. *Plant Methods* **2018**, *14* (1), 1–12.
(27) Nitta, S.; Yamamoto, A.; Kurita, M.; Arakawa, R.; Kawasaki, H. Gold-Decorated Titania Nanotube Arrays as Dual-Functional Platform for Surface-Enhanced Raman Spectroscopy and Surface-Assisted Laser Desorption/Ionization Mass Spectrometry. *ACS Appl. Mater. Interfaces* **2014**, *6* (11), 8387–8395.
(28) Oshima, Y.; Shinzawa, H.; Takenaka, T.; Furihata, C.; Sato, H. Discrimination Analysis of Human Lung Cancer Cells Associated with Histological Type and Malignancy Using Raman Spectroscopy. *J. Biomed. Opt.* **2010**, *15* (1), 017009.
(29) Cebeci, D.; Alam, A.; Wang, P.; Pinal, R.; Ben-amotz, D. Photobleaching Profile of Raman Peaks and Fluorescence Background. *Eur. Pharm. Rev.* **2017**, *22* (6), 18–21.
(30) Baranska, M. *Optical Spectroscopy and Computational Methods in Biology and Medicine*; 2014.
(31) Rolinger, L.; Matthias, R. A Critical Review of Recent Trends , and a Future Perspective of Optical Spectroscopy as PAT in Biopharmaceutical Downstream Processing. **2020**, 2047–2064.
(32) Lasch, P. Spectral Pre-Processing for Biomedical Vibrational Spectroscopy and Microspectroscopic Imaging. *Chemom. Intell. Lab. Syst.* **2012**, *117*, 100–114.
(33) Vidal, M.; Amigo, J. M. Pre-Processing of Hyperspectral Images. Essential Steps before Image Analysis. *Chemom. Intell. Lab. Syst.* **2012**, *117*, 138–148.
(34) Gautam, R.; Vanga, S.; Ariese, F.; Umapathy, S. Review of Multidimensional Data Processing Approaches for Raman and Infrared Spectroscopy. *EPJ Tech. Instrum.* **2015**, *2* (1).
(35) Lanekoff, I.; Heath, B. S.; Liyu, A.; Thomas, M.; Carson, J. P.; Laskin, J. Automated Platform for High-Resolution Tissue Imaging Using Nanospray Desorption Electrospray Ionization

Mass Spectrometry. *Anal. Chem.* **2012**, *84* (19), 8351–8356.

(36) Kompauer, M.; Heiles, S.; Spengler, B. Atmospheric Pressure MALDI Mass Spectrometry Imaging of Tissues and Cells at 1.4-Mm Lateral Resolution. *Nat. Methods* **2016**, *14* (1), 90–96.

(37) Wang, H. A. O.; Grolimund, D.; Giesen, C.; Borca, C. N.; Shaw-Stewart, J. R. H.; Bodenmiller, B.; Günther, D. Fast Chemical Imaging at High Spatial Resolution by Laser Ablation Inductively Coupled Plasma Mass Spectrometry. *Anal. Chem.* **2013**, *85* (21), 10107–10116.

(38) Limbeck, A.; Galler, P.; Bonta, M.; Bauer, G.; Nischkauer, W.; Vanhaecke, F. Recent Advances in Quantitative LA-ICP-MS Analysis: Challenges and Solutions in the Life Sciences and Environmental Chemistry ABC Highlights: Authored by Rising Stars and Top Experts. *Anal. Bioanal. Chem.* **2015**, *407* (22), 6593–6617.

(39) Kollmer, F.; Paul, W.; Krehl, M.; Niehuis, E. Ultra High Spatial Resolution SIMS with Cluster Ions - Approaching the Physical Limits. *Surf. Interface Anal.* **2013**, *45* (1), 312–314.

(40) Yoon, S.; Lee, T. G. Biological Tissue Sample Preparation for Time-of-Flight Secondary Ion Mass Spectrometry (ToF–SIMS) Imaging. *Nano Converg.* **2018**, *5* (1).

(41) Bertasa, M.; Possenti, E.; Botteon, A.; Conti, C.; Sansonetti, A.; Fontana, R.; Striova, J.; Sali, D. Close to the Diffraction Limit in High Resolution ATR FTIR Mapping: Demonstration on Micrometric Multi-Layered Art Systems. *Analyst* **2017**, *142* (24), 4801–4811.

(42) Kazarian, S. G.; Chan, K. L. A. Applications of ATR-FTIR Spectroscopic Imaging to Biomedical Samples. *Biochim. Biophys. Acta - Biomembr.* **2006**, *1758* (7), 858–867.

(43) Baig, N. F.; Dunham, S. J. B.; Morales-Soto, N.; Shrout, J. D. .; Sweedler, J. V. .; Bohn, P. W. Multimodal Chemical Imaging of Molecular Messengers in Emerging Pseudomonas Aeruginosa Bacterial Communities. *Analyst* **2015**, *140* (19), 6544–6552.

(44) Bergholt, M. S.; Serio, A.; McKenzie, J. S.; Boyd, A.; Soares, R. F.; Tillner, J.; Chiappini, C.; Wu, V.; Dannhorn, A.; Takats, Z.; Williams, A.; Stevens, M. M. Correlated Heterospectral Lipidomics for Biomolecular Profiling of Remyelination in Multiple Sclerosis. *ACS Cent. Sci.* **2018**, *4* (1), 39–51.

(45) Bocklitz, T. W.; Crecelius, A. C.; Matthäus, C.; Tarcea, N.; von Eggeling, F.; Schmitt, M.; Schubert, U. S.; Popp, J. Deeper Understanding of Biological Tissue: Quantitative Correlation of MALDI-TOF and Raman Imaging. *Anal. Chem.* **2013**, *85* (22), 10829–10834.

(46) Bocklitz, T.; Bräutigam, K.; Urbanek, A.; Hoffmann, F.; von Eggeling, F.; Ernst, G.; Schmitt, M.; Schubert, U.; Guntinas-Lichius, O.; Popp, J. Novel Workflow for Combining Raman Spectroscopy and MALDI-MSI for Tissue Based Studies. *Anal. Bioanal. Chem.* **2015**, *407*

(26), 7865–7873.

(47) Bradshaw, R.; Wolstenholme, R.; Ferguson, L. S.; Sammon, C.; Mader, K.; Claude, E.; Blackledge, R. D.; Clench, M. R.; Francese, S. Spectroscopic Imaging Based Approach for Condom Identification in Condom Contaminated Fingermarks. *Analyst* **2013**, *138* (9), 2546.

(48) Lanni, E. J.; Masyuko, R. N.; Driscoll, C. M.; Dunham, S. J. B.; Shrout, J. D.; Bohn, P. W.; Sweedler, J. V. Correlated Imaging with C 60 -SIMS and Confocal Raman Microscopy: Visualization of Cell-Scale Molecular Distributions in Bacterial Biofilms. *Anal. Chem.* **2014**, *86* (21), 10885–10891.

(49) Li, Z.; Chu, L.-Q.; Sweedler, J. V.; Bohn, P. W. Spatial Correlation of Confocal Raman Scattering and Secondary Ion Mass Spectrometric Molecular Images of Lignocellulosic Materials. *Anal. Chem.* **2010**, *82* (7), 2608–2611.

(50) Morales-Soto, N.; Dunham, S. J. B.; Baig, N. F.; Ellis, J. F.; Madukoma, C. S.; Bohn, P. W.; Sweedler, J. V.; Shrout, J. D. Spatially Dependent Alkyl Quinolone Signaling Responses to Antibiotics in Pseudomonas Aeruginosa Swarms. *J. Biol. Chem.* **2018**, *293* (24), 9544–9552.

(51) Neumann, E. K.; Comi, T. J.; Spegazzini, N.; Mitchell, J. W.; Rubakhin, S. S.; Gillette, M. U.; Bhargava, R.; Sweedler, J. V. Multimodal Chemical Analysis of the Brain by High Mass Resolution Mass Spectrometry and Infrared Spectroscopic Imaging. *Anal. Chem.* **2018**, *90* (19), 11572–11580.

(52) Petit, V. W.; Réfrégiers, M.; Guettier, C.; Jamme, F.; Sebanayakam, K.; Brunelle, A.; Laprévote, O.; Dumas, P.; Le Naour, F. Multimodal Spectroscopy Combining Time-of-Flight-Secondary Ion Mass Spectrometry, Synchrotron-FT-IR, and Synchrotron-UV Microspectroscopies on the Same Tissue Section. *Anal. Chem.* **2010**, *82* (9), 3963–3968.

(53) Ryabchykov, O.; Popp, J.; Bocklitz, T. Fusion of MALDI Spectrometric Imaging and Raman Spectroscopic Data for the Analysis of Biological Samples. *Front. Chem.* **2018**, *6*, 1–10.
